# CPT1A drives cisplatin resistance via acetylation‑dependent activation of DRP1 and mitochondrial fission in small cell lung cancer

**DOI:** 10.1038/s41419-026-08868-x

**Published:** 2026-05-28

**Authors:** Kuanbing Chen, Shi Zong, Kefeng Li, Li Yu

**Affiliations:** 1 Department of Thoracic Surgery, Shengjing Hospital of China Medical University, Shenyang, Liaoning China; 2 Department of Thoracic surgery, Shenyang Tenth People’s Hospital/Shenyang Chest Hospital, Shenyang, Liaoning China; 3 Center for Artificial Intelligence-Driven Drug Discovery, Faculty of Applied Sciences, Macao Polytechnic University, Macao, China; 4 Department of Oncology, Shengjing Hospital of China Medical University, Shenyang, Liaoning China

**Keywords:** Cancer, Molecular biology

## Abstract

Cisplatin resistance represents a major clinical challenge in small-cell lung cancer (SCLC), yet the underlying metabolic adaptations remain poorly understood. Here, we identify a novel regulatory axis centered on the fatty acid oxidation (FAO) enzyme carnitine palmitoyltransferase 1 A (*CPT1A*) that governs mitochondrial dynamics to drive chemoresistance. In cisplatin-resistant SCLC, *CPT1A* is markedly upregulated and undergoes functional acetylation. This modified CPT1A not only sustains cellular bioenergetics and redox balance through enhanced FAO but also directly recruits dynamin-related protein 1 (DRP1) to mitochondria. By facilitating DRP1-dependent mitochondrial fission, CPT1A orchestrates a metabolic adaptation that confers a survival advantage. Genetic or pharmacological inhibition of *CPT1A* reversed this phenotype, impairing mitochondrial fission, depleting energy stores, and resensitizing resistant cells to cisplatin. In vivo, targeting *CPT1A* markedly suppressed tumor growth and restored cisplatin sensitivity. Our results uncover an acetylated CPT1A-DRP1 axis as a critical metabolic vulnerability in cisplatin-resistant SCLC, providing a compelling therapeutic strategy to overcome treatment failure.

## Introduction

Lung cancer remains the most common cause of cancer-related deaths globally, with an estimated 2.2 million new cases and 1.8 million deaths annually. Small-cell lung cancer (SCLC), a highly aggressive subtype, constitutes approximately 15% of all lung cancer cases and is characterized by rapid growth and early dissemination. Despite the initial sensitivity to chemotherapy, most SCLC patients experience relapses due to the development of drug resistance, especially to cisplatin, the cornerstone of treatment regimens [1, 2].

Cisplatin exerts its cytotoxic effects by forming DNA adducts that disrupt DNA replication and transcription, ultimately inducing apoptosis. However, tumor cells often develop mechanisms to evade this cytotoxicity, including enhanced DNA repair, drug efflux, and metabolic reprogramming [3, 4]. Among these, metabolic adaptation, particularly through fatty acid oxidation (FAO), has garnered attention as a critical driver of chemoresistance. FAO serves as a primary energy source under metabolic stress, providing ATP and reducing equivalents to sustain tumor growth and survival [5].

Carnitine palmitoyltransferase 1 A (*CPT1A*) is a rate-limiting enzyme in FAO that facilitates the transport of long-chain fatty acids into mitochondria for oxidation. Elevated *CPT1A* expression has been implicated in various cancers, promoting tumor progression and resistance to therapy by supporting energy production and redox balance [6, 7]. However, the role of CPT1A in cisplatin resistance, particularly its post-translational modifications, remains underexplored.

Mitochondrial dynamics, which include the processes of fission and fusion, are essential for maintaining cellular homeostasis. Dynamin-related protein 1 (DRP1) is a key mediator of mitochondrial fission, and its activation is associated with enhanced mitochondrial fragmentation, metabolic reprogramming, and tumor progression [8, 9]. Recent studies suggest that DRP1 activity is modulated by post-translational modifications such as phosphorylation and acetylation, which influence its localization and function [10, 11].

In this study, we hypothesize that CPT1A drives cisplatin resistance in SCLC via a novel mechanism: by regulating the acetylation status of DRP1, leading to dysregulated mitochondrial fission. We investigated the interplay between CPT1A and DRP1, elucidating how this axis drives metabolic reprogramming and mitochondrial remodeling. By targeting the CPT1A-DRP1 axis, we aim to provide a novel therapeutic strategy to overcome cisplatin resistance in SCLC.

## Materials and methods

### Cell lines and reagents

Human SCLC cell lines (H526, H69) and their cisplatin-resistant derivatives (H526R, H69R) were obtained from FUHENG BIOLOGY (Changsha, China). All cell lines were authenticated by short tandem repeat (STR) profiling within the last year and tested negative for mycoplasma contamination using a PCR-based detection kit (Sigma-Aldrich, USA) prior to experiments. All cells were cultured in RPMI-1640 medium supplemented with 10% fetal bovine serum (FBS, Gibco), 100 U/mL penicillin, and 100 µg/mL streptomycin at 37 °C in a 5% CO2 atmosphere.

### Lentiviral transduction and stable cell line generation

Lentiviral shRNA and overexpression vectors for *CPT1A* and *DRP1* were constructed by Tsingke Biotechnology. Control vectors included non-targeting shRNA (shNC) and empty vector. The shRNA target sequences were: shNC, 5’-UUCUCCGAACGUGUCACGUTT-3’; shCPT1A, 5’-GCATGTTTATTTCATTCTAAG-3’; shDRP1, 5’-ACUAUUGAAGGAACUGCAAA-3’. Lentiviruses were packaged in HEK293T cells using psPAX2/pMD2.G plasmids and Lipofectamine 3000. Supernatants were harvested at 48 h, filtered (0.45 μm), and concentrated. H526R and H69R cells were transduced (8 μg/mL polybrene) and selected with puromycin (2 μg/mL, 7–10 days). Efficiency was validated by qRT-PCR and Western blot.

### RNA isolation, qPCR, and primer design

Total RNA was extracted using the TRIzol reagent (Thermo Fisher Scientific) according to the manufacturer’s protocol. RNA was reverse transcribed into cDNA using the iScript™ cDNA Synthesis Kit (Bio-Rad). Quantitative PCR (qPCR) was performed using SYBR Green Master Mix (Thermo Fisher Scientific) on an ABI 7500 Real-Time PCR system. The primers used are as follows:

*CPT1A* forward: 5′-AGTCCTGGTTTCTGGGGAGT-3′,

*CPT1A* reverse: 5′-TCAGGTCAGCAAGAACTCCT-3′,

*DRP1* forward: 5′-CCCTGCCTTCTTATCTGGGT-3′,

*DRP1* reverse: 5′-GTGACGTTGCTTCTGTTGGT-3′,

*β-actin* forward: 5′-AGAGCTACGAGCTGCCTGAC-3′,

*β-actin* reverse: 5′-AGCACTGTGTTGGCGTACAG-3′.

### Western blot analysis

Protein lysates were prepared using RIPA buffer supplemented with protease and phosphatase inhibitors (Roche). Protein concentration was determined using a BCA Protein Assay Kit (Thermo Fisher Scientific). Proteins were separated by SDS-PAGE and transferred to PVDF membranes (Millipore). Membranes were blocked in 5% non-fat milk and incubated overnight at 4 °C with the following primary antibodies: Anti-CPT1A (Abcam, ab128568, 1:1000), Anti-DRP1 (Cell Signaling Technology, #8570, 1:1000), Anti-p-DRP1 (Ser616) (Cell Signaling Technology, #3455, 1:1000), Anti-acetyl-lysine (Cell Signaling Technology, #9441, 1:1000), Anti-β-actin (Sigma-Aldrich, A1978, 1:5000), Secondary HRP-conjugated antibodies (1:3000) were used, and signals were detected using ECL reagent (Thermo Fisher Scientific).

### Mitochondrial network visualization

To visualize the mitochondrial network, cells were stained with MitoTracker Deep Red FM (100 nM; Thermo Fisher Scientific; 30 min; 37 °C), fixed with 4% paraformaldehyde, and permeabilized. After blocking, cells were incubated with an anti-DRP1 (1:300; ab184247; Abcam) followed by an Alexa Fluor 488-conjugated secondary antibody. Nuclei were stained with DAPI. Confocal z-stack images (Zeiss LSM 880, 63× oil objective) were acquired. Mitochondrial morphology (fragmented, intermediate, or elongated) was assessed and quantified in at least 50 cells per condition from three independent experiments.

### Immunohistochemistry (IHC) and immunofluorescence

Paraffin-embedded tissue sections were deparaffinized and rehydrated. Antigen retrieval was performed using citrate buffer (pH 6.0). Sections were blocked with 5% BSA and incubated overnight at 4 °C with primary antibodies: Anti-CPT1A (Abcam, ab128568, 1:200), Anti-Ki-67 (Cell Signaling Technology, 9027, 1:200), Anti-CD36 (Abcam, ab133625, 1:200). Formalin-fixed, paraffin-embedded tumor tissues were sectioned (4 µm) and subjected to IHC analysis as described above. For IHC, sections were treated with HRP-conjugated secondary antibodies and developed using DAB substrate. For immunofluorescence, Alexa Fluor-conjugated secondary antibodies were used, and nuclei were counterstained with DAPI. Images were captured using a Nikon Eclipse fluorescence microscope. CPT1A expression was quantified using ImageJ software, and staining intensity was scored by two independent pathologists.

### Oxygen consumption rate (OCR) and extracellular acidification rate (ECAR) measurement

OCR and ECAR were measured using the Seahorse XF Analyzer (Agilent Technologies, USA). Cells were seeded in XF96 cell culture microplates at an optimized density (1.5 × 10⁴ cells per well for H526/H526R; 2 × 10⁴ cells per well for H69/H69R) and incubated overnight to allow attachment. On the day of assay, culture medium was replaced with Seahorse XF RPMI medium (pH 7.4) supplemented with 10 mM glucose, 1 mM pyruvate, and 2 mM glutamine, and cells were incubated at 37 °C without CO₂ for 1 h prior to measurement.

For mitochondrial stress tests, OCR was measured under basal conditions and following sequential injection of 1 oligomycin (1 µM), FCCP (1 µM), and rotenone/antimycin A (0.5 µM). For FAO-dependent respiration assays, cells were incubated overnight in substrate-limited medium (DMEM with 0.5 mM glucose, 1 mM glutamine, 0.5% FBS) prior to the assay. On the day of measurement, medium was replaced with FAO assay medium (KHB buffer supplemented with 2.5 mM glucose, 0.5 mM carnitine, and 5 mM HEPES) and incubated for 30 min. To assess FAO dependency, parallel wells were treated with 50 µM etomoxir (Sigma-Aldrich), a specific CPT1 inhibitor that blocks mitochondrial fatty acid uptake, or vehicle control for 15 min prior to the addition of 200 µM palmitate-BSA or BSA control. OCR was then measured following the same injection protocol.

All OCR and ECAR values were normalized to cell number using a Hoechst 33342-based fluorescence assay (Invitrogen) or parallel cell counting. Each condition was assayed in at least quintuplicate wells, and experiments were repeated three times independently.

### Lactate assay and cell proliferation assays

Cell culture supernatants were collected, and lactate levels were measured using a lactate assay kit (Sigma-Aldrich) according to the manufacturer’s protocol. Absorbance was measured at 450 nm. Cell proliferation was assessed using the cell counting kit-8 (CCK-8) assay. Cells were seeded in 96-well plates (5 × 10^3^ cells/well) and treated with cisplatin (20 µM) continuously. At the indicated time points (12, 24, 36, 48, 72, and 96 h after cisplatin addition), 10 µL of CCK-8 reagent was added per well and incubated for 2 h at 37 °C. Absorbance was measured at 450 nm using a microplate reader. Each condition was assayed in at least triplicate wells, and experiments were independently repeated three times.

### Bromodeoxyuridine (BrdU) incorporation and clonogenic assay

Cells grown on coverslips were pulsed with 10 µM BrdU for 2 h. Cells were then fixed (4% PFA), permeabilized (0.3% Triton X-100), and DNA was denatured (2 M HCl) for 30 min at 37 °C. After blocking, samples were incubated with an anti-BrdU (Santa Cruz Biotechnology, Inc., sc-32323, 1:200) and an Alexa Fluor-conjugated secondary antibody. Nuclei were counterstained with DAPI. The percentage of BrdU-positive cells was quantified from fluorescence microscope images by counting at least five random fields per coverslip. For clonogenic assay, cells were seeded in 6-well plates (500 cells/well) and treated with cisplatin (10 µM) for 14 days, with the culture medium being refreshed every 3–4 day. Colonies were fixed with 4% paraformaldehyde, stained with crystal violet, and counted manually. Each condition was performed in triplicate wells, and experiments were independently repeated three times.

### ATP and redox measurements

Intracellular ATP levels were measured using an ATP assay kit (Promega) following the manufacturer’s instructions. Redox balance was assessed by measuring NADPH/NADP+ and GSH/GSSG ratios using commercially available kits (Abcam). Each condition was performed in triplicate wells, and experiments were independently repeated three times.

### Immunoprecipitation (IP) and immunofluorescence (IF)

For IP, cells or tissue lysates were prepared in RIPA buffer supplemented with protease and phosphatase inhibitors. Protein lysates (500 µg) were incubated with 2 µg of primary antibodies overnight at 4 °C with gentle rotation, followed by incubation with 30 µL of protein G agarose beads for 4 h at 4 °C. Beads were washed five times with lysis buffer, and bound proteins were eluted by boiling in SDS loading buffer. Input controls (10% of lysate) were included in all experiments.

For acetylation analysis of DRP1, immunoprecipitation was performed using anti-acetyl-lysine antibodies (Abcam, ab80178, 1:1000), followed by immunoblotting with anti-DRP1 antibody to detect acetylated DRP1 levels. Total cell lysates (Input) were probed for DRP1 as loading controls.

For co-immunoprecipitation (Co-IP) to detect protein-protein interaction, reciprocal IPs were performed: cell lysates were immunoprecipitated with either anti-CPT1A or anti-DRP1 antibodies, followed by immunoblotting with the reciprocal antibody. IgG was used as a negative control.

For IF, cells on glass coverslips were incubated with MitoTracker Red (Thermo Fisher, 100 nM) for 30 min at 37 °C to label mitochondria. Cells were then fixed with 4% paraformaldehyde for 15 min, permeabilized with 0.1% Triton X-100 for 10 min, and blocked with 5% BSA for 1 h. Samples were incubated with anti-DRP1 antibody (1:200) overnight at 4 °C, followed by Alexa Fluor 488-conjugated secondary antibody (1:500) for 1 h. Nuclei were counterstained with DAPI. Coverslips were mounted using antifade mounting medium. Images were acquired using a confocal microscope (Zeiss LSM 880) and analyzed with ImageJ. Colocalization was quantified using Pearson’s correlation coefficient.

### In vivo tumor xenograft model

All animal experiments were conducted in strict accordance with institutional guidelines and were approved by the Institutional Animal Care and Use Committee (IACUC) of [ZJCLA-IACUC-24020017]. For the xenograft study, female nude mice (6-8 weeks old) were subcutaneously injected with 5 × 10^6^ H526R cells that had been stably transduced with sh*CPT1A* or oe*CPT1A* lentiviral constructs (*n* = 6 mice per group). Mice were randomized into groups and treated with cisplatin (5 mg/kg, i.p.) or vehicle control twice weekly for four weeks. Tumor volume was measured biweekly using calipers, and tissues were analyzed by immunohistochemistry for CPT1A, CD36, and Ki-67 expression. From cell line injection to the completion of all analyses, the researchers remained blinded. Investigators were blinded to group allocation during tumor measurements and data analysis.

### Statistical analysis

All experiments were conducted in triplicate. Results are expressed as mean ± SD. Statistical analyses were performed using GraphPad Prism 9.0. One-way ANOVA followed by Tukey’s multiple comparisons test was used for group comparisons. A *P* value < 0.05 was considered significant.

## Results

### CPT1A expression is elevated in cisplatin-resistant SCLC cells and correlates with enhanced fatty acid oxidation

**Fig. 1:**
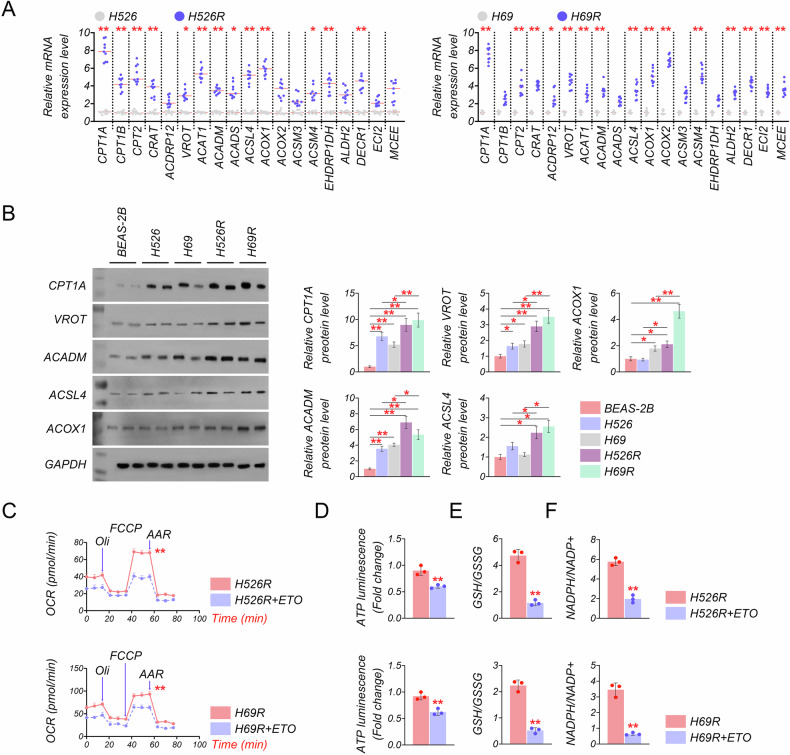
CPT1A is upregulated and promotes FAO in cisplatin-resistant SCLC cells. **A** qPCR analysis of FAO-related genes in cisplatin-resistant H526R and H69R cells compared to their parental counterparts. **B** Western blot validation of FAO enzyme protein expression (CPT1A, VROT, ACADM, ACSL4, and ACOX1) in the same cell lines. **C** Basal oxygen consumption rate (OCR) measured by Seahorse assay in H526R and H69R cells treated with Etomoxir (ETO, 50 µM) or vehicle control. **D** Intracellular ATP levels in cisplatin-resistant H526R and H69R cells treated with the CPT1A inhibitor etomoxir (ETO, 50 µM) or vehicle control for 24 hours. **E**, **F** Quantification of NADPH/NADP+ ratio and GSH/GSSG ratio under the same conditions. Data are presented as mean ± SD from three independent experiments. *n* = 3, *p < 0.05, **p < 0.01.

To determine the role of fatty acid metabolism in cisplatin resistance, we compared the expression of FAO-related genes between parental and cisplatin-resistant SCLC cell lines. A marked upregulation of *CPT1A* was observed in both H526R and H69R resistant cells compared to their parental counterparts, a finding that was consistent at both the mRNA and protein levels (Fig. 1A, B). This elevation was not limited to CPT1A; other key FAO genes, including *VROT, ACADM, ACSL4, and ACOX1*, were also significantly increased in the resistant cell lines, suggesting a broad transcriptional activation of the FAO pathway (Fig. 1A, B).

To determine whether this transcriptional upregulation translates into a functional metabolic dependency, we assessed FAO activity in H526R and H69R cells. Seahorse flux analysis revealed that resistant cells are highly reliant on FAO for their basal respiration, as treatment with the CPT1 inhibitor etomoxir (ETO, 50 µM) significantly suppressed the basal OCR in both H526R and H69R cells (Fig. 1C). This metabolic reliance has critical functional consequences. Inhibiting FAO not only depleted intracellular ATP levels (Fig. 1D) but also severely compromised the cellular redox balance, as evidenced by significantly reduced GSH/GSSG and NADPH/NADP+ ratios (Fig. 1E, F). These results demonstrate that cisplatin-resistant SCLC cells undergo a metabolic rewiring that renders them exquisitely dependent on FAO to sustain both their bioenergetic and antioxidant defenses.

### CPT1A is essential for the survival and metabolic fitness of cisplatin-resistant SCLC cells

**Fig. 2:**
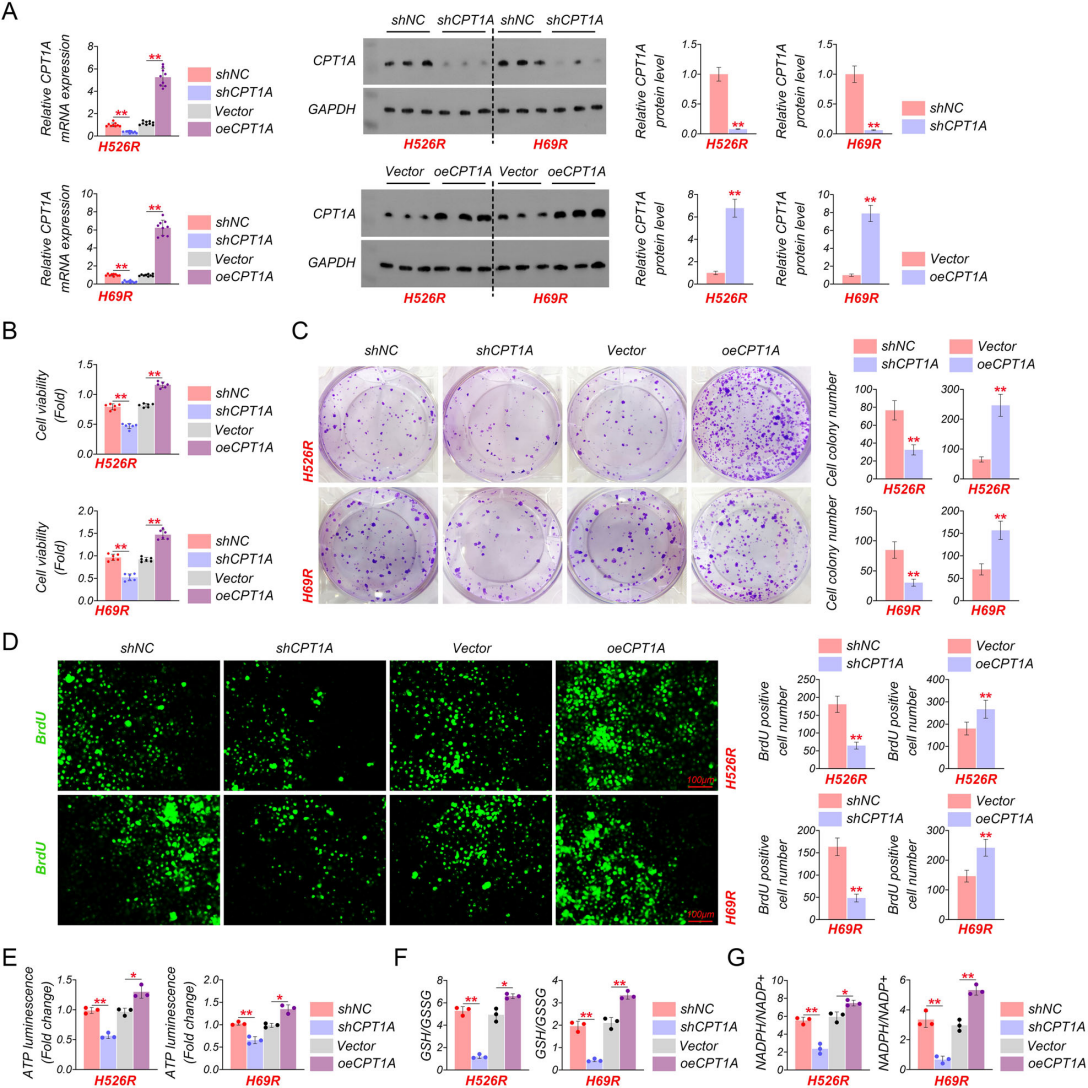
CPT1A is Essential for the Survival and Metabolic Fitness of Cisplatin-resistant SCLC Cells. **A** Validation of *CPT1A* knockdown (sh*CPT1A)* and overexpression (oe*CPT1A*) efficiency by qRT-PCR and Western blotting. **B** Cell proliferation curves at 12, 24, 36, 48, 72, and 96 h measured by CCK-8 assay following CPT1A modulation in H526R and H69R cells. **C** Representative images and quantification of colony formation assays. **D** BrdU incorporation assay following CPT1A modulation. **E** Measurement of intracellular ATP levels after CPT1A modulation. **F**, **G** Quantification of NADPH/NADP+ and GSH/GSSG ratios in corresponding cells. *n* = 3, *p < 0.05, **p < 0.01.

To investigate the functional role of *CPT1A* in cisplatin-resistant SCLC cells, we first modulated its expression in H526R and H69R cells (Fig. 2A). Functionally, *CPT1A* proved critical for long-term proliferative capacity. Knockdown of *CPT1A* significantly suppressed cell viability over 4-day period, whereas its *CPT1A* overexpression lconferred a marked growth advantage (Fig. 2B). This dependency extended to clonogenic survival, which was severely impaired by *CPT1A* silencing and enhanced by its overexpression (Fig. 2C). Consistently, BrdU incorporation assays revealed that *CPT1A* knockdown significantly reduced DNA synthesis, while *CPT1A* overexpression enhanced it (Fig. 2D), indicating that *CPT1A* is required for maintaining proliferative capacity in cisplatin-resistant SCLC cells. Mechanistically, the pro-survival function of *CPT1A* is linked to its role in maintaining cellular energy and redox balance. *CPT1A* knockdown in resistant cells triggered a severe bioenergetic crisis, evidenced by decreased ATP production and compromised redox homeostasis, as indicated by reduced GSH/GSSG and NADPH/NADP+ ratios (Fig. 2E–G).

To determine if this reliance on *CPT1A* was specific to the resistant phenotype, we performed parallel experiments in the parental, non-resistant H526 and H69 cells. While *CPT1A* knockdown in parental cells also reduced colony formation and ATP levels, the effects were substantially attenuated compared to those observed in their resistant counterparts (Figure S1A–G). This direct comparison reveals that cisplatin-resistant cells are more vulnerable to CPT1A inhibition than their parental counterparts. Collectively, these results demonstrate that CPT1A is critically required for the proliferation and clonogenic survival of cisplatin-resistant SCLC cells, primarily by sustaining energy productio.

### CPT1A regulates metabolic reprogramming and enhances tumor progression

**Fig. 3:**
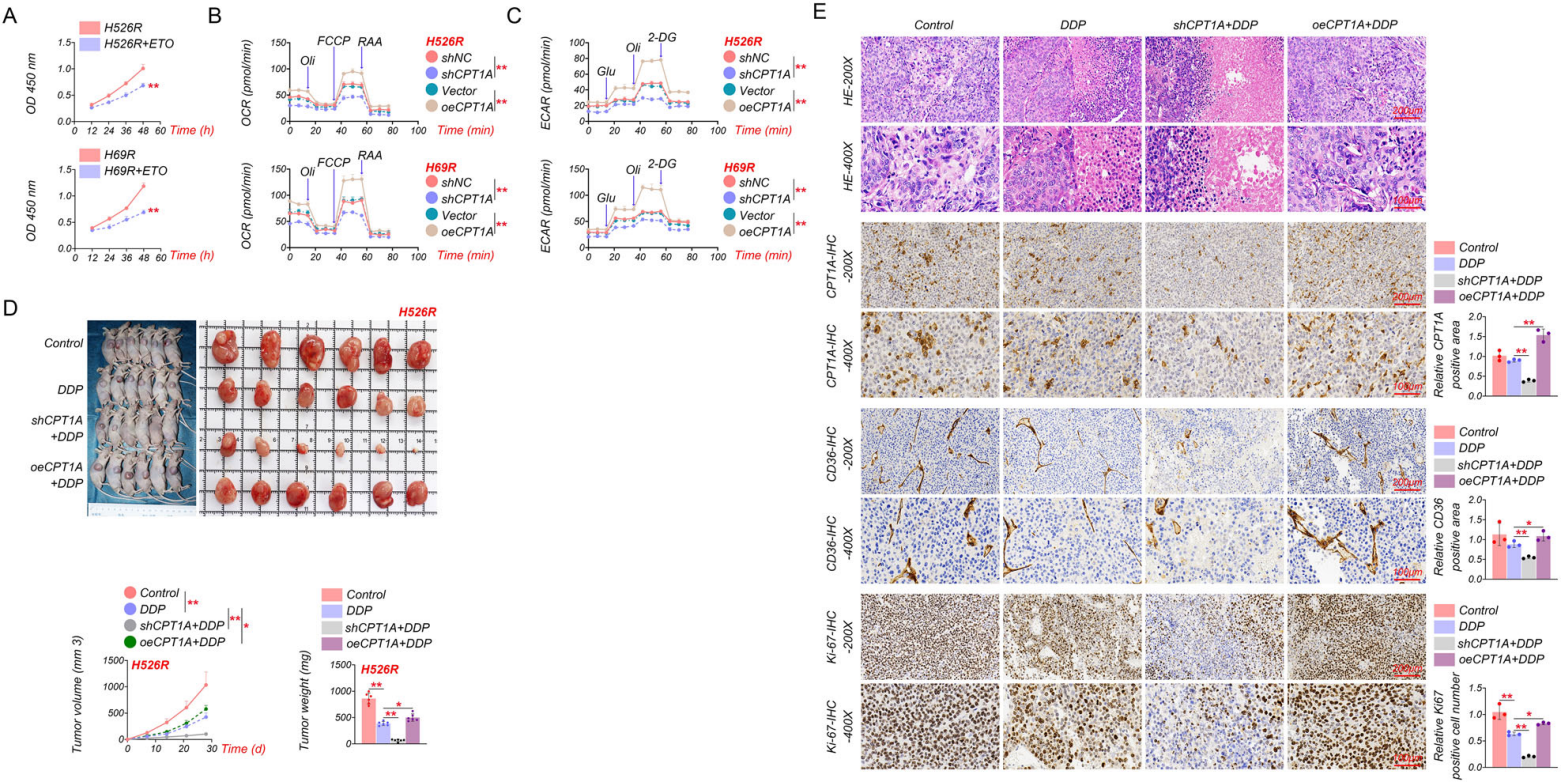
CPT1A promotes metabolic reprogramming and tumor growth in cisplatin-resistant cells. **A** Cell proliferation curves at 12, 24, 36, and 48 h measured by CCK-8 assay after ETO treatment in H526R and H69R cells. **B**, **C** Basal oxygen consumption rate (OCR) and extracellular acidification rate (ECAR) assessed by Seahorse XF analysis following sh*CPT1A* or oe*CPT1A* transfection. **D** Lactate production quantified in corresponding groups. **E** OCR analysis under different treatments (control, ETO, palmitate, palmitate+ETO) in *CPT1A*-modulated cells. **D** Tumor growth curves and tumor weights in nude mice injected with H526R cells (shNC, DDP, sh*CPT1A* + DDP, oe*CPT1A* + DDP groups). **E** Representative H&E staining and immunohistochemical staining for CPT1A, CD36 (a fatty acid uptake receptor), and Ki-67 in xenograft tumors. Scale bars: 200 μm. *n* = 6, *p < 0.05, **p < 0.01.

To determine the metabolic basis of cisplatin resistance, we examined the effect of *CPT1A* manipulation on cell proliferation and core metabolic pathways. ETO treatment significantly reduced cell proliferation in H526R and H69R cells, indicating a reliance on FAO for growth (Fig. 3A). We next examined how *CPT1A* modulates mitochondrial respiration. Knockdown of *CPT1A* (sh*CPT1A*) markedly reduced both basal and maximal oxygen consumption rate (OCR) in resistant cells, whereas its overexpression (oe*CPT1A*) enhanced these parameters (Fig. 3B), demonstrating that *CPT1A* is a key determinant of mitochondrial respiratory capacity. Interestingly, the metabolic influence of *CPT1A* extended beyond oxidative phosphorylation. *CPT1A* knockdown decreased both ECAR and intracellular lactate levels, while its overexpression had the opposite effect (Fig. 3C and Figure S2A). This coordinated regulation suggests that *CPT1A* supports a broader metabolic reprogramming, enhancing both oxidative and glycolytic flux to sustain the heightened bioenergetic demands of cisplatin-resistant SCLC cells.

We next investigated how *CPT1A* modulates mitochondrial oxidative phosphorylation (OXPHOS) and whether targeting this pathway could be exploited therapeutically. Using Seahorse analysis, we assessed OCR in *CPT1A*-modulated cells under various pharmacological conditions (Figure S2B). Consistent with our earlier findings, basal OCR was significantly higher in oe*CPT1A* cells than in sh*CPT1A* cells under control conditions. In oe*CPT1A* cells, palmitate (PAL) monotherapy did not alter basal OCR but significantly suppressed maximal OCR, an effect that was more pronounced than with ETO alone. Strikingly, combined PAL and ETO treatment synergistically impaired both basal and maximal OCR, but only in oe*CPT1A* cells; in sh*CPT1A* cells, neither monotherapy nor combination treatment had any effect, confirming that this synergy is strictly *CPT1A*-dependent.

We next evaluated the therapeutic potential of targeting *CPT1A* in vivo using a xenograft mouse model. Consistent with our in vitro findings, *CPT1A* modulation profoundly influenced tumor growth in combination with cisplatin (DDP) treatment. While DDP alone reduced tumor parameters compared to controls, tumors with *CPT1A* knockdown (sh*CPT1A* + DDP) were the smallest, whereas those with *CPT1A* overexpression (oe*CPT1A* + DDP) were larger (Fig. 3D). Immunohistochemical analyses of the tumors revealed a coordinated metabolic program underlying this differential growth. The sh*CPT1A* + DDP group exhibited significant downregulation of CPT1A, the fatty acid transporter CD36 (a key supplier of substrates for FAO), and the proliferation marker Ki-67; conversely, all three markers were upregulated in the oe*CPT1A* + DDP group (Fig. 3E). The coordinated changes in CPT1A and CD36 suggest a concomitant suppression of both fatty acid uptake and oxidation, thereby impairing the FAO-driven survival pathway in these tumors. Together, these results demonstrate that CPT1A promotes tumor progression by integrating fatty acid uptake and oxidation to fuel the bioenergetic and proliferative demands of cisplatin-resistant SCLC.

### CPT1A regulates mitochondrial dynamics in cisplatin-resistant cells

**Fig. 4:**
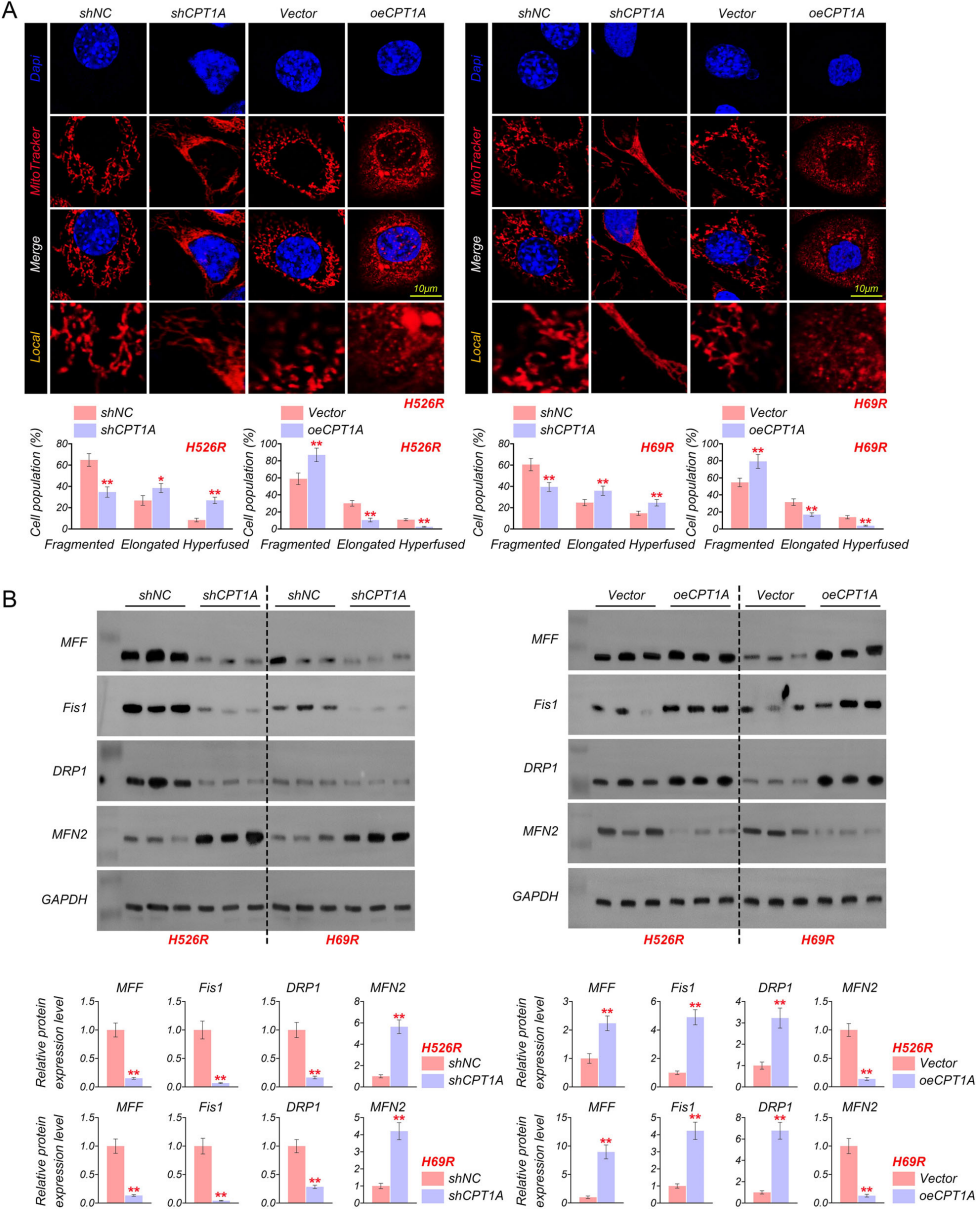
CPT1A controls mitochondrial morphology by modulating fission and fusion balance. **A** Representative images of MitoTracker Deep Red FM staining showing mitochondrial network morphology. Right panel shows quantitative analysis classifying mitochondrial morphology as fragmented, intermediate, or elongated. Scale bar: 10 μm. **B** Western blotting of mitochondrial fission (MFF, Fis1, DRP1) and fusion (MFN2) markers following *CPT1A* knockdown or overexpression. *n* = 3, *p < 0.05, **p < 0.01.

To evaluate the impact of CPT1A on mitochondrial morphology, we first visualized mitochondria in CPT1A-modulated H526R and H69R cells using MitoTracker™ Red CMXRos staining. Confocal microscopy revealed that *CPT1A* knockdown (sh*CPT1A*) cells exhibited elongated, interconnected mitochondrial networks, indicative of a fused state. In contrast, *CPT1A*-overexpressing (oe*CPT1A*) cells displayed punctate and fragmented mitochondria, characteristic of enhanced fission (Fig. 4A).

Consistent with this morphological shift, western blot analysis showed that *CPT1A* expression coordinately regulates the molecular machinery governing mitochondrial dynamics. In oe*CPT1A* cells, fission-promoting factors (MFF, DRP1, Fis1) were significantly upregulated, while the fusion protein MFN2 was downregulated; an opposite expression pattern was observed in sh*CPT1A* cells (Fig. 4B). Taken together, these data demonstrate that CPT1A drives mitochondrial fragmentation in cisplatin-resistant SCLC cells by shifting the balance of mitochondrial dynamics regulators toward a pro-fission state.

### CPT1A regulates mitochondrial dynamics through DRP1

We next investigated the functional relationship between CPT1A and DRP1 in cisplatin-resistant SCLC cells. Successful modulation of *DRP1* and *CPT1A* expression was verified by qRT-PCR and western blot (Figure S3A, B). Notably, DRP1 overexpression increased CPT1A levels, while DRP1 knockdown decreased them; in contrast, CPT1A silencing had only a minimal effect on DRP1 mRNA, suggesting that CPT1A acts predominantly downstream of DRP1 in this regulatory axis. Functionally, *DRP1* overexpression enhanced cell viability, whereas knockdown of either *DRP1* or *CPT1A* impaired growth (Figure S3C). Importantly, silencing *CPT1A* in *DRP1*-overexpressing cells partially abrogated the pro-proliferative effect of *DRP1*, indicating that *CPT1A* is a critical downstream mediator of *DRP1*-driven proliferation in cisplatin-resistant SCLC cells.

To determine whether the CPT1A-DRP1 axis coordinately regulates mitochondrial function, we assessed multiple metabolic parameters. Consistent with their roles in promoting proliferation, oe*DRP1* increased ATP production, whereas knockdown of either *CPT1A* or *DRP1* reduced ATP levels by more than 50% (Figure S3D). This cooperative effect extended to redox balance, as GSH/GSSG and NADPH/NADP+ ratios were significantly elevated in oe*DRP1* cells but dramatically reduced following *CPT1A* or *DRP1* knockdown (Figure S3E, F). These results indicate that the CPT1A-DRP1 axis is critical for maintaining antioxidant defense. Mitochondrial respiration capacity followed a similar pattern. Basal OCR was significantly higher in oe*DRP1* cells and markedly suppressed in sh*CPT1A* and sh*DRP1* cells (Figure S3G). Furthermore, both maximal respiration and spare respiratory capacity (Figure S3H–I), key indicators of mitochondrial functional reserve, were enhanced by oe*DRP1* but reduced by sh*CPT1A* or sh*DRP1*. Collectively, these findings indicate that CPT1A and DRP1 functionally cooperate to sustain mitochondrial bioenergetics and redox homeostasis. This cooperation reveals that DRP1-driven mitochondrial fission is essential for maintaining the metabolic reserves that support proliferation in cisplatin-resistant SCLC cells.

### CPT1A stabilizes DRP1 via acetylation

**Fig. 5:**
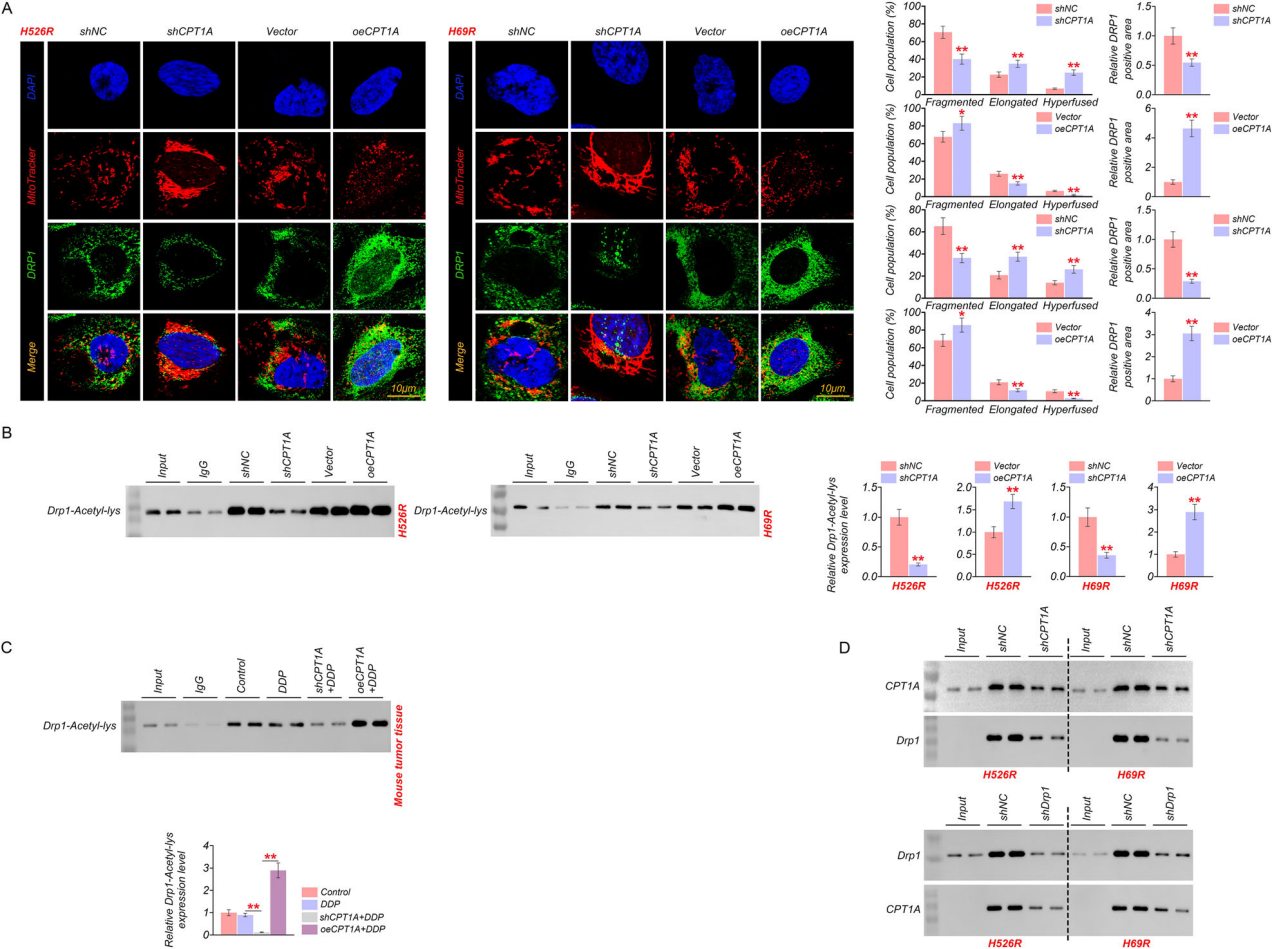
CPT1A regulates DRP1 acetylation and mitochondrial localization. **A** Representative immunofluorescence staining of DRP1 (green), mitochondria (MitoTracker Red) and nuclei (DAPI, blue) in H526R and H69R cells under three conditions: control (shNC/Vector), *CPT1A* knockdown (sh*CPT1A*), and *CPT1A* overexpression (oe*CPT1A*). Right panel shows quantification of DRP1 mitochondrial recruitment and mitochondrial morphology, presented as the proportion of cells with fragmented, elongated, or hyperfused mitochondria. Scale bar: 10 μm. **B** CPT1A enhances DRP1 acetylation. Immunoprecipitation (IP) assay was performed using an anti‑acetyl‑lysine antibody, followed by Western blot analysis with a DRP1 antibody, to assess DRP1 acetylation levels in shNC, sh*CPT1A*, and oe*CPT1A* cells. Total cell lysates (Input) and the immunoprecipitated complexes (IP: Ac-K) are shown. The bar graph (right) presents the quantitative analysis of acetylated DRP1 normalized to total DRP1 in the input lanes. **C** CPT1A modulates DRP1 acetylation in vivo and in response to cisplatin. IP assay using an anti-acetyl-lysine antibody was performed on lysates from mouse xenograft tumor tissues treated under four conditions: Control (vehicle), DDP (cisplatin), DDP + sh*CPT1A*, and DDP + oe*CPT1A*. The Western blot was probed for DRP1 to assess its acetylation status. Quantification (lower panel) shows that *CPT1A* knockdown reduced, while *CPT1A* overexpression enhanced, DRP1 acetylation levels in the presence of DDP. **D** CPT1A physically interacts with DRP1. Reciprocal co-immunoprecipitation (Co-IP) assays in H526R and H69R cells confirm the direct protein-protein interaction. Upper panel: IP was performed using an anti-DRP1 antibody, followed by Western blotting for CPT1A. *CPT1A* knockdown reduced the CPT1A signal co-precipitated with DRP1. Lower panel: IP was performed using an anti-CPT1A antibody, followed by Western blotting for DRP1. *DRP1* knockdown reduced the DRP1 signal co-precipitated with CPT1A. *n* = 3, **p < 0.01.

Studies have shown that the activity and mitochondrial localization of DRP1 are precisely regulated by various post-translational modifications (PTMs), including phosphorylation, ubiquitination, and SUMOylation, which collectively determine its pro-mitochondrial fission function [12, 13]. It is particularly noteworthy that acetylation, as an emerging regulatory modification, has been reported to enhance DRP1 activity and lead to excessive mitochondrial fission under stress conditions such as hypoxia/ischemia [14]. However, in tumor cells with metabolic reprogramming, the upstream signals driving DRP1 acetylation and its physiological significance remain unclear. Our research reveals the central role of CPT1A, a key enzyme in fatty acid oxidation, in this process. First, immunofluorescence experiments confirmed that CPT1A expression levels directly regulate the mitochondrial recruitment of DRP1. *CPT1A* overexpressing enhanced DRP1 colocalization with mitochondrial markers (MitoTracker), while *CPT1A* knocking down diminished it (Fig. 5A). Mechanistically, CPT1A modulated DRP1acetylation, as overexpression of *CPT1A* significantly enhanced DRP1 acetylation, and knockdown reduced it (Fig. 5B). This regulatory role was further corroborated in vivo, where *CPT1A* knockdown reversed the DDP-induced DRP1 acetylation and *CPT1A* overexpression enhanced this effect (Fig. 5C).

To determine whether CPT1A exerts this effect through direct interaction, we performed co-immunoprecipitation assays. CPT1A knockdown reduced the CPT1A signal in DRP1 immunoprecipitates, and conversely, DRP1 knockdown reduced the DRP1 signal in CPT1A immunoprecipitates (Fig. 5D). These results confirm a direct protein-protein interaction between CPT1A and DRP1, providing a molecular basis for CPT1A-mediated regulation of DRP1 acetylation and mitochondrial dynamics in cisplatin-resistant SCLC cells.

### Inhibition of DRP1 phosphorylation modulates DRP1 acetylation and mitochondrial fission

**Fig. 6:**
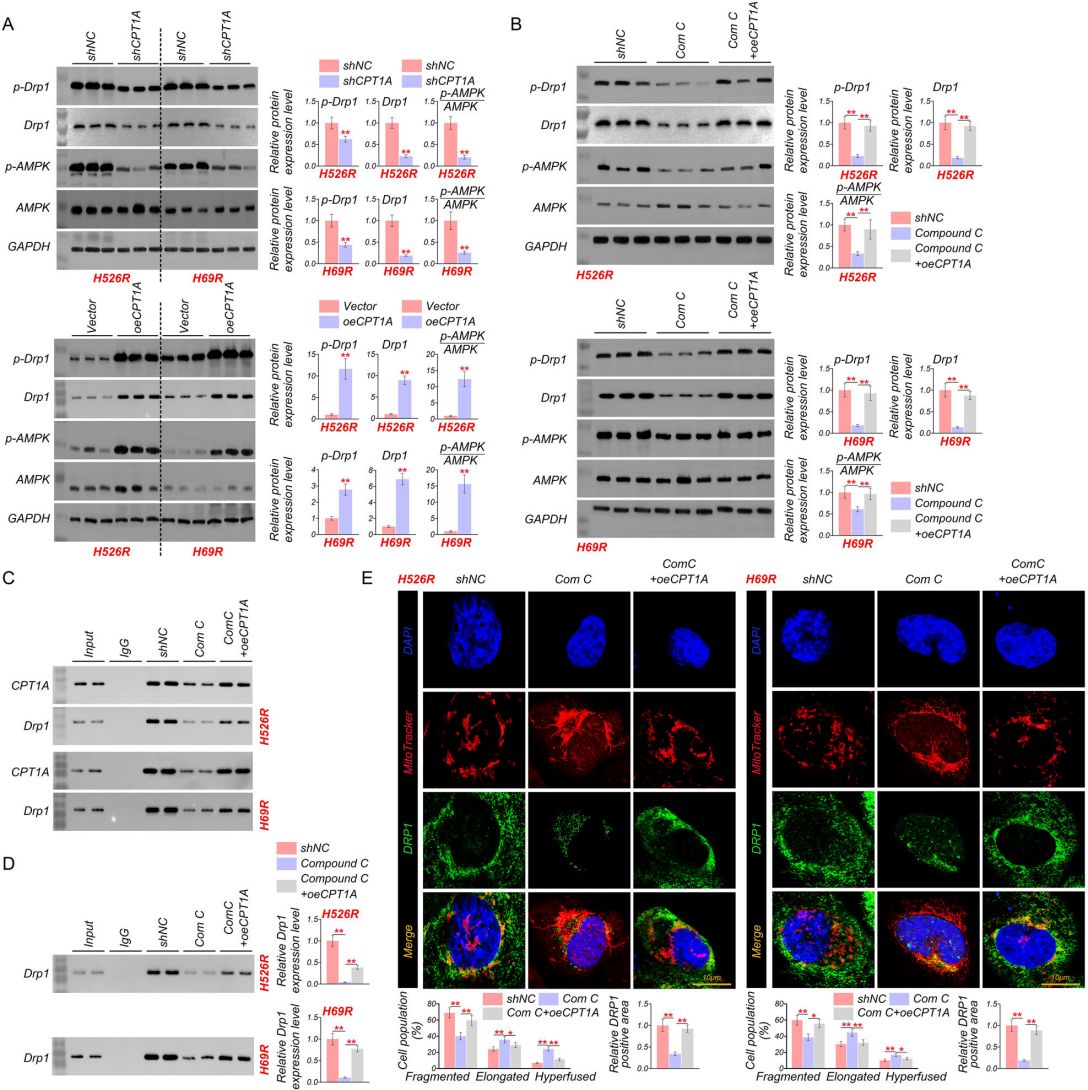
AMPK signaling mediates CPT1A-driven DRP1 phosphorylation and mitochondrial fission. **A** Western blot analysis of phosphorylated (p-) and total levels of AMPK and DRP1 after *CPT1A* knockdown or overexpression. **B** Effects of AMPK inhibition by Compound C (10 µM, 24 h) and rescue by *CPT1A* overexpression on p-AMPK and p-DRP1 levels. **C** Co-IP assay showing that Compound C treatment weakened the interaction between CPT1A and DRP1, while CPT1A overexpression partially restored and enhanced this association. **D** Immunoprecipitation (IP) assay and its quantitative analysis demonstrating that Compound C treatment suppressed DRP1 protein activity, and this suppression was partially reversed by CPT1A overexpression. **E** Representative immunofluorescence images depicting mitochondrial DRP1 (green) and mitochondrial network (MitoTracker Red) after indicated treatments. The lower panel shows quantitative analysis of mitochondrial elongation/fragmentation. Scale bar: 10 μm. *n* = 3, **p < 0.01.

Given that AMPK-mediated phosphorylation influences DRP1 function and its susceptibility to acetylation [15], we asked whether CPT1A regulates DRP1 through this phospho-dependent pathway. CPT1A expression directly correlated with the activation of this axis, as overexpression of *CPT1A* elevated levels of phospho-DRP1 (Ser616) and phospho-AMPK while *CPT1A* knockdown diminished them (Fig. 6A, B). Critically, the AMPK inhibitor Compound C abrogated the CPT1A-induced increase in p-DRP1, confirming that AMPK activity is required for the phosphorylation of DRP1 promoted by CPT1A (Fig. 6A, B).

The functional interaction between CPT1A and DRP1 was further probed by co-immunoprecipitation. Inhibition of AMPK phosphorylation with Compound C reduced the physical association between CPT1A and DRP1. This attenuated interaction was partially rescued by *CPT1A* overexpression, suggesting that CPT1A helps maintain the DRP1 complex in an AMPK-phosphorylation-dependent manner (Fig. 6C). Accordingly, immunoprecipitation and quantitative analysis indicated that Compound C suppressed DRP1 activity, an effect that was partially reversed by concurrent *CPT1A* overexpression (Fig. 6D). Finally, to assess the functional consequences on mitochondrial dynamics, we analyzed mitochondrial morphology. Compound C treatment induced a shift from fragmented to elongated/over-fused mitochondria, indicative of reduced fission activity. *CPT1A* overexpression counteracted this change, significantly decreasing the proportion of elongated mitochondria and increasing DRP1-positive puncta at mitochondrial sites (Fig. 6E). This regulatory axis was conserved across both H526R and H69R cell lines.

### Clinical relevance of CPT1A and DRP1 in cisplatin-resistant lung cancer

Immunohistochemical analysis of human tissue samples revealed a progressive, parallel upregulation of CPT1A and DRP1 expression from normal lung tissue to small-cell lung cancer (SCLC) and, most notably, to cisplatin-resistant SCLC (Figure S4). The expression levels were lowest in normal tissue, moderate in SCLC, and highest in the resistant tumors. A positive correlation between CPT1A and DRP1 protein levels supports the clinical relevance of this axis and suggests its association with the cisplatin-resistant phenotype. These findings nominate the CPT1A-DRP1 regulatory axis as a potential biomarker and a therapeutic target worthy of further investigation in treatment-resistant lung cancer.

## Discussion

Cisplatin resistance remains a major challenge in the clinical management of SCLC. Our study identifies a novel post-translational mechanism underpinning this resistance, centered on the functional crosstalk between metabolic reprogramming and mitochondrial dynamics. We demonstrate that CPT1A undergoes functional acetylation, a modification critical for its non-canonical role in recruiting DRP1 to mitochondria. This promotes excessive fission and drives a metabolic state conducive to chemoresistance. These findings significantly advance the paradigm that metabolic and organellar adaptations are intrinsically linked in treatment-refractory cancers.

The oncogenic role and therapeutic potential of CPT1A have been extensively documented across various malignancies, including ovarian cancer [16], esophageal squamous cell carcinoma [17], glioblastoma [18], and colon cancer [12], where it drives key malignant phenotypes such as cell cycle progression, anchorage-independent growth, and chemoresistance. Building upon this established context, our study now identifies CPT1A as a critical transcriptional driver specifically in cisplatin-resistant SCLC. We further provide mechanistic evidence that its upregulation is essential for maintaining bioenergetic and redox homeostasis under cisplatin pressure. These findings not only extend the pathogenic role of CPT1A to a new and aggressive cancer type but also clarify its precise function within the chemoresistant niche, moving beyond correlation to establish causation.

A fundamental mechanism of chemoresistance lines in the metabolic reprogramming of cancer cells, which enables survival under therapeutic stress [19, 20]. A hallmark of this adaptation is the elevation of FAO, a pathway critical for meeting the heightened bioenergetic and redox demands of aggressive tumors [5]. In cisplatin-resistant SCLC cells, this is driven by the transcriptional upregulation of CPT1A. Consequently, these cells exhibit enhanced FAO flux, elevated ATP production, and a robust antioxidant capacity, establishing CPT1A as a functional driver that directly links metabolic reprogramming to the resistant phenotype.

This metabolic shift must be coordinated with cellular architecture to be fully effective. Mitochondrial dynamics, particularly DRP1-mediated fission, are essential for such adaptation and are themselves strongly associated with therapy resistance and poor prognosis [8, 10, 11]. A key unresolved question has been how metabolic signals, such as enhanced FAO, are transduced into pro-fission mitochondrial remodeling. Our work bridges this gap by revealing CPT1A as a direct molecular partner for DRP1. The acetylation-dependent enhancement of their interaction provides a precise mechanism for coupling elevated lipid catabolism to mitochondrial fragmentation. This “metabolite-sensor-to-organelle-shaper” axis represents a significant conceptual advance over models where metabolism and dynamics are regulated in parallel.

Our data reveal that CPT1A, beyond its metabolic function, acts as a direct molecular partner for DRP1. Our co-immunoprecipitation data confirm their physical interaction, which is enhanced by DRP1 acetylation. This interaction facilitates the stable recruitment of DRP1 to the mitochondrial outer membrane, leading to a hyperfragmented network. Consequently, genetic or pharmacological inhibition of CPT1A or DRP1 disrupted mitochondrial architecture, crippled oxidative phosphorylation, and resensitized cells to cisplatin. This establishes a direct, actionable axis where a metabolic enzyme directly commands an organelle-remodeling protein.

We further delineated the regulatory hierarchy within this axis. Our data show that AMPK-mediated phosphorylation of DRP1 is a prerequisite for its subsequent its acetylation and stable mitochondrial association. This places AMPK activity upstream, integrating cellular energy stress signals with the CPT1A-DRP1 machinery to execute mitochondrial fission. Inhibition of AMPK destabilized the CPT1A-DRP1 complex and phenocopied the effects of CPT1A/DRP1 knockdown, confirming that this signaling cascade is indispensable for the pro-fission, pro-survival adaptation. This intricate regulation aligns with reports positioning AMPK as a nexus between metabolism and dynamics [21, 22] and provides a mechanistic explanation for how stress signaling converges on mitochondrial morphology in resistant cells.

The efficacy of *CPT1A* knockdown in restoring cisplatin sensitivity in vivo, coupled with the co-upregulation of CPT1A and DRP1 in patient-derived resistant tumors, underscores its clinical relevance. Mechanistically, the resultant tumors exhibited not only diminished proliferation (Ki-67) but also a marked reduction in CD36 expression. CD36 is a key fatty acid translocase responsible for cellular uptake of long-chain fatty acids, the primary substrates for CPT1A and subsequent FAO [23]. Its downregulation upon CPT1A targeting confirms a profound disruption of the upstream fuel supply for this metabolic pathway. This coordinated suppression of both CPT1A and its substrate importer (CD36) highlights a tightly regulated feedforward loop in treatment-resistant tumors. Importantly, this CPT1A-CD36 functional link is not isolated to SCLC; a recent study in intrahepatic cholangiocarcinoma demonstrated that the CPT1A inhibitor etomoxir exerted its anti-tumor effect precisely by controlling CD36-mediated fatty acid metabolism, thereby inhibiting cancer progression [24]. This parallel finding across cancer types strongly reinforces the generality and therapeutic relevance of targeting the upstream fatty acid uptake and utilization network.

Collectively, these findings position CPT1A not merely as a metabolic enzyme but as a pivotal signaling node that transduces metabolic demands into structural mitochondrial changes. Therefore, pharmacological inhibition of CPT1A emerges as a rational therapeutic strategy to simultaneously cripple two pillars of chemoresistance: metabolic flexibility and adaptive mitochondrial fission. Future therapeutic development should explore the synergy between FAO inhibitors, DRP1 inhibitors, and standard platinum-based regimens [25, 26]. Such a combinatorial approach could effectively dismantle the integrated survival network of resistant SCLC cells.

In conclusion, we have elucidated a previously unrecognized acetylated CPT1A-DRP1 axis that drives cisplatin resistance in SCLC by integrating metabolic fueling with mitochondrial fragmentation. Targeting this axis presents a promising strategy to overcome chemoresistance in SCLC, with potential applicability to other malignancies reliant on FAO and dynamic mitochondrial remodeling.

## Supplementary information

Figure S1

Figure S2

Figure S3

Figure S4

supplementary figure legends

WB original

## Data Availability

Data sharing is not applicable to this article as no new data were created or analyzed in this study.
