# Supplementary figures and images for: CPT1A drives cisplatin resistance via acetylation‑dependent activation of DRP1 and mitochondrial fission in small cell lung cancer
Source: Cell Death Dis. 2026 May 28;17(1):661. doi: 10.1038/s41419-026-08868-x (PMC13407905; doi:10.1038/s41419-026-08868-x)

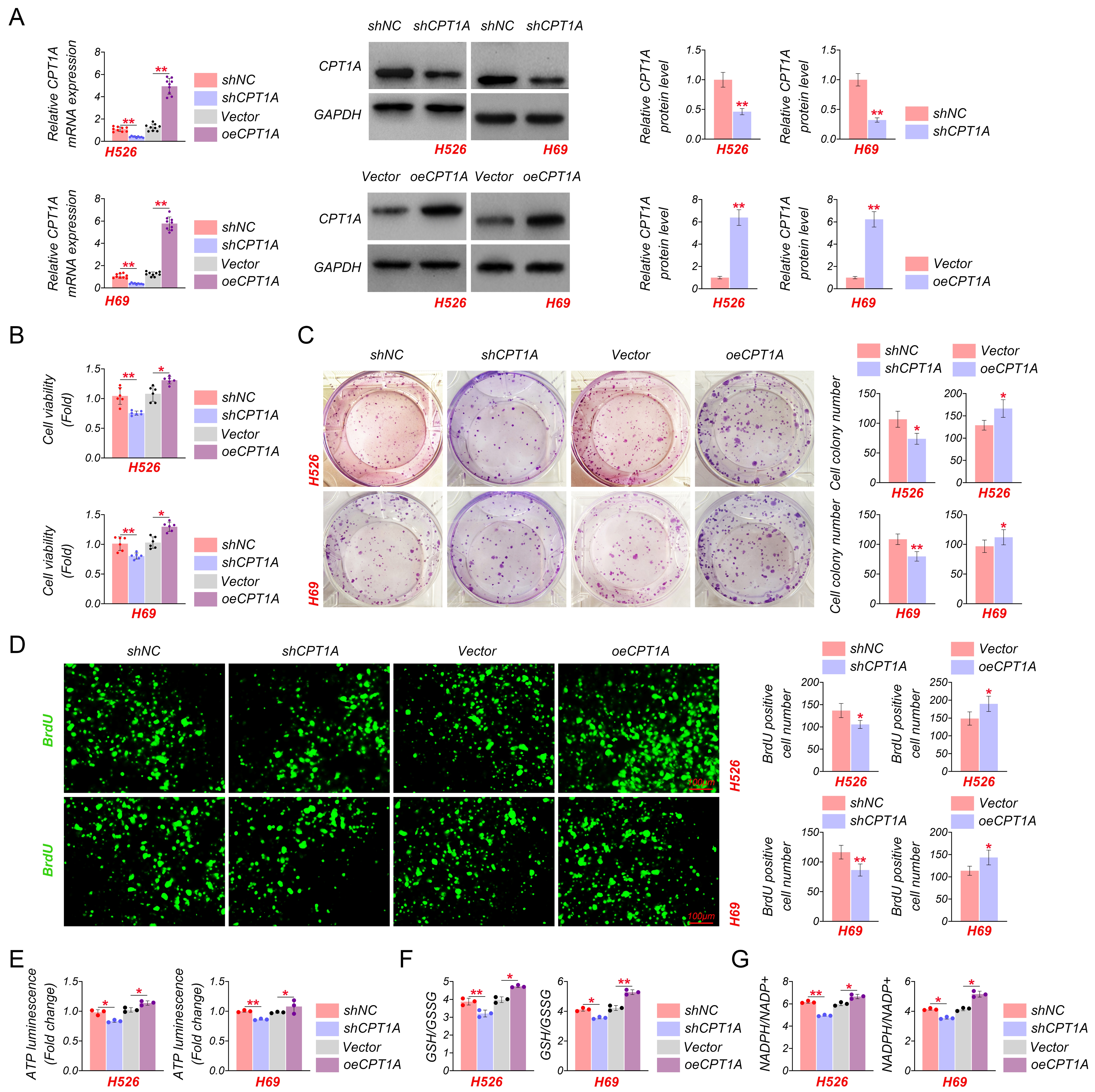

Supplement: Supplementary file 1 — Figure S1 [file 41419_2026_8868_MOESM1_ESM.jpg]

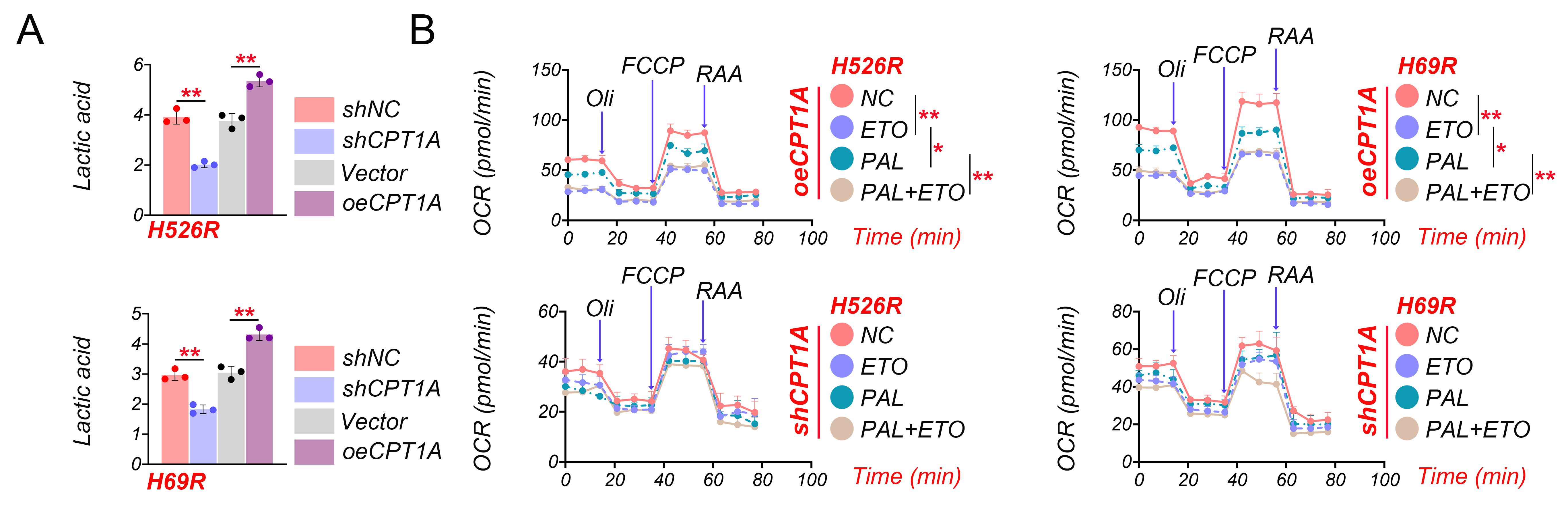

Supplement: Supplementary file 2 — Figure S2 [file 41419_2026_8868_MOESM2_ESM.jpg]

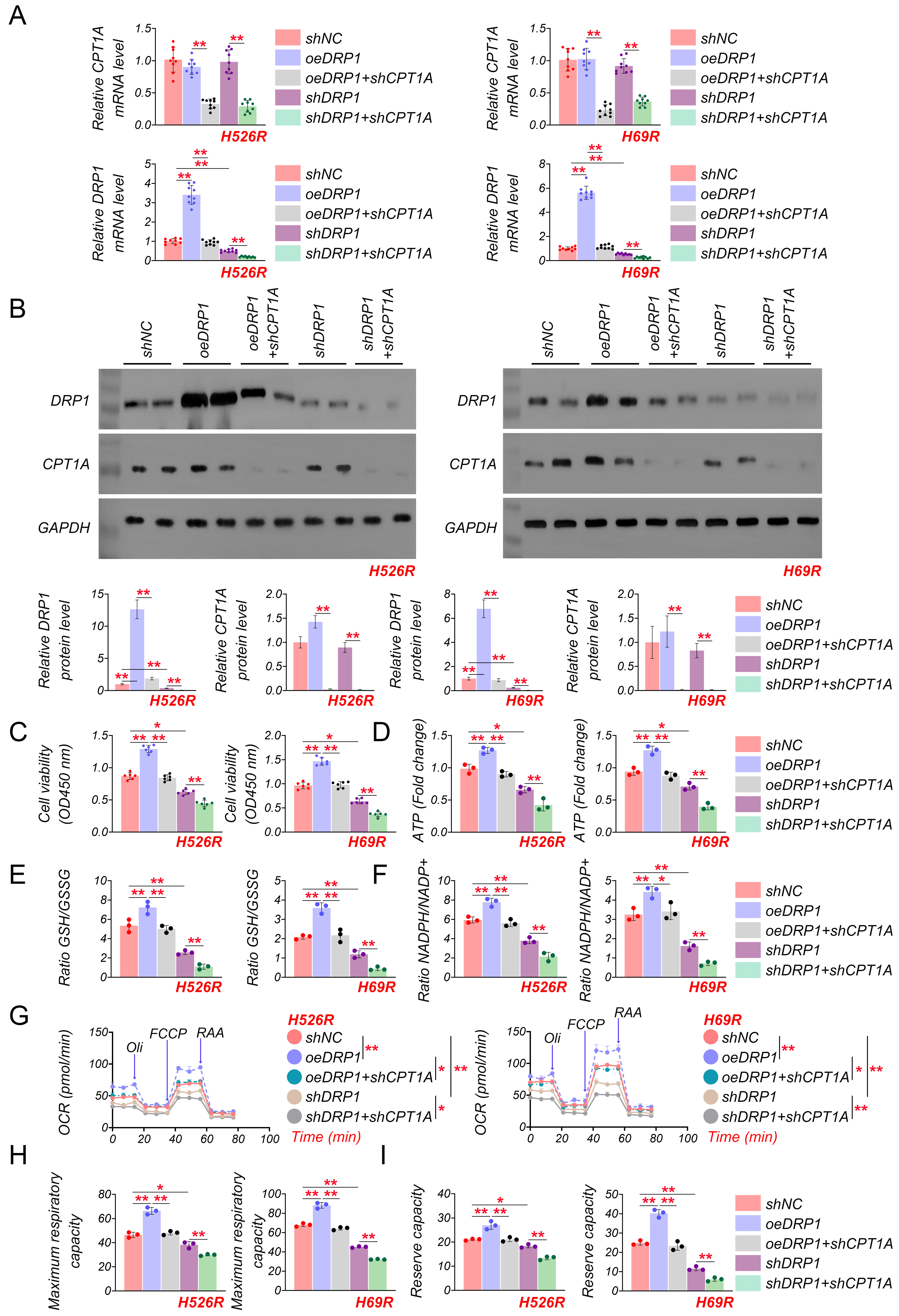

Supplement: Supplementary file 3 — Figure S3 [file 41419_2026_8868_MOESM3_ESM.jpg]

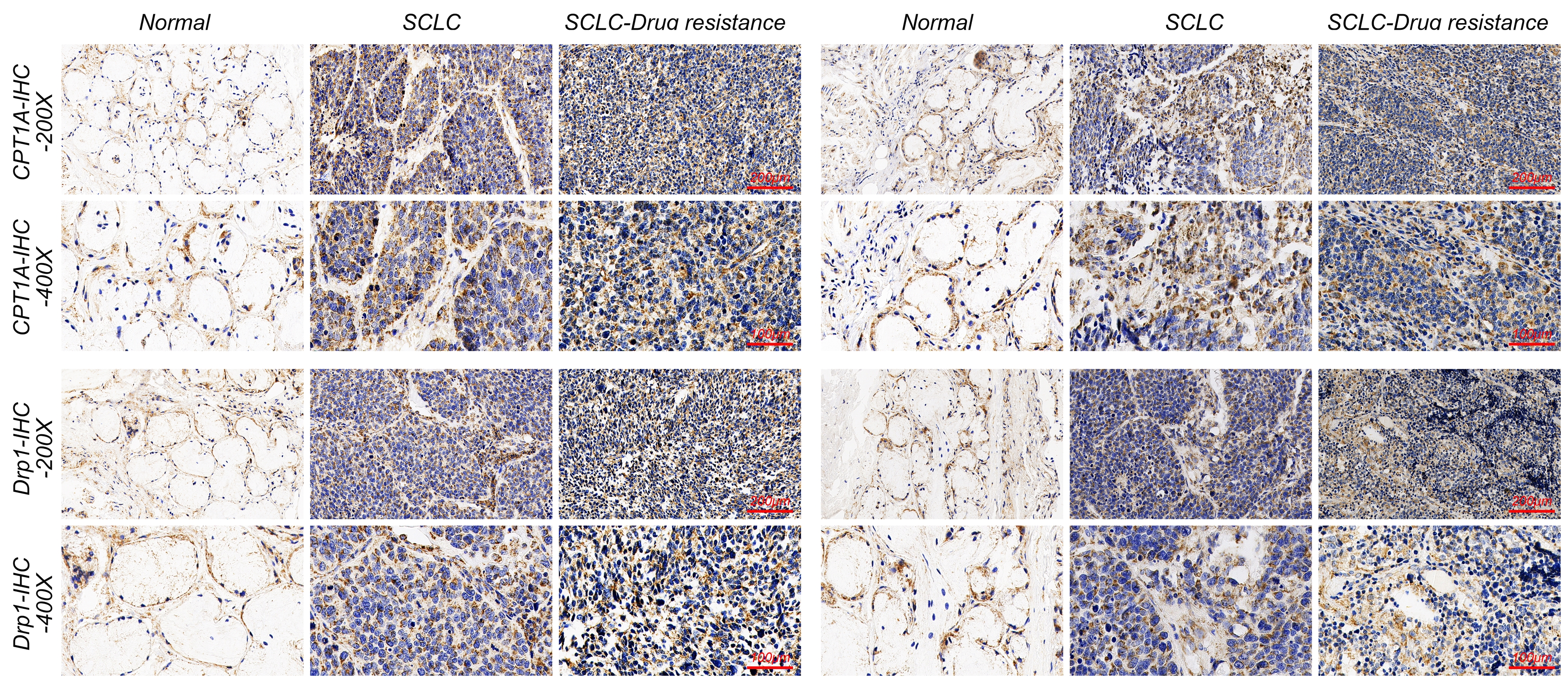

Supplement: Supplementary file 4 — Figure S4 [file 41419_2026_8868_MOESM4_ESM.jpg]
