# Supplementary material for: CPT1A drives cisplatin resistance via acetylation‑dependent activation of DRP1 and mitochondrial fission in small cell lung cancer
Source: Cell Death Dis. 2026 May 28;17(1):661. doi: 10.1038/s41419-026-08868-x (PMC13407905; doi:10.1038/s41419-026-08868-x)
Supplement: Supplementary file 5 — supplementary figure legends [file 41419_2026_8868_MOESM5_ESM.doc]

**Supplementary Figure S1. Functional validation of CPT1A modulation in cisplatin-resistant SCLC cells.**

1. Validation of CPT1A knockdown (sh*CPT1A*) and overexpression (oe*CPT1A*) efficiency by quantitative RT-PCR (mRNA) and western blotting (protein) in H526R and H69R cells. (B) Cell proliferation measured by CCK-8 assay at indicated time points following CPT1A modulation. (C) Representative images and quantification of colony formation assays. (D) DNA synthesis assessed by BrdU incorporation assay. Scale bar: 100 μm. (E) Intracellular ATP levels measured after CPT1A modulation. (F, G) Cellular redox status evaluated by the GSH/GSSG ratio (F) and the NADPH/NADP+ ratio (G). n =3, ^*^*p* < 0.05, ^**^*p* < 0.01.

**Supplementary Figure S2. CPT1A promotes metabolic reprogramming and tumor growth in cisplatin-resistant cells.** (A) Lactate production quantified in corresponding groups. (B) OCR analysis under different treatments (control, ETO, palmitate, palmitate+ETO) in *CPT1A*-modulated cells. n =3, ^*^*p* < 0.05, ^**^*p* < 0.01.

**Supplementary Figure S3. CPT1A cooperates with DRP1 to maintain mitochondrial bioenergetics.** (A) Quantitative RT-PCR analysis of DRP1 and *CPT1A* mRNA levels in H526R and H69R cells transfected with shNC, oe*DRP1*, oeDRP1+sh*CPT1A*, sh*DRP1*, or sh*DRP1*+sh*CPT1A*. (B) Representative Western blot images and quantification of DRP1 and CPT1A protein expression normalized to GAPDH. (C) Cell viability assessed by CCK-8 assay. (D) Intracellular ATP levels measured using a luminescence-based ATP assay. (E-F) Cellular redox status assessed by GSH/GSSG (E) and NADPH/NADP+ (F) ratios. (G) Basal oxygen consumption rate (OCR) profiles measured using a Seahorse XF Analyzer. (H) Quantification of maximum respiratory capacity. (I) Quantification of spare (reserve) respiratory capacity. n =3, ^*^*p* < 0.05, ^**^*p* < 0.01.

**Supplementary Figure S4. CPT1A and DRP1 expression are elevated in cisplatin-resistant SCLC patient tissues.**
Representative immunohistochemical staining of CPT1A and DRP1 in human normal lung tissue, cisplatin-sensitive SCLC tumors, and cisplatin-resistant SCLC tumors. Scale bar: 100 μm.
