# Supplementary material for: CPT1A drives cisplatin resistance via acetylation‑dependent activation of DRP1 and mitochondrial fission in small cell lung cancer
Source: Cell Death Dis. 2026 May 28;17(1):661. doi: 10.1038/s41419-026-08868-x (PMC13407905; doi:10.1038/s41419-026-08868-x)
Supplement: Supplementary file 6 — WB original [file 41419_2026_8868_MOESM6_ESM.pdf]

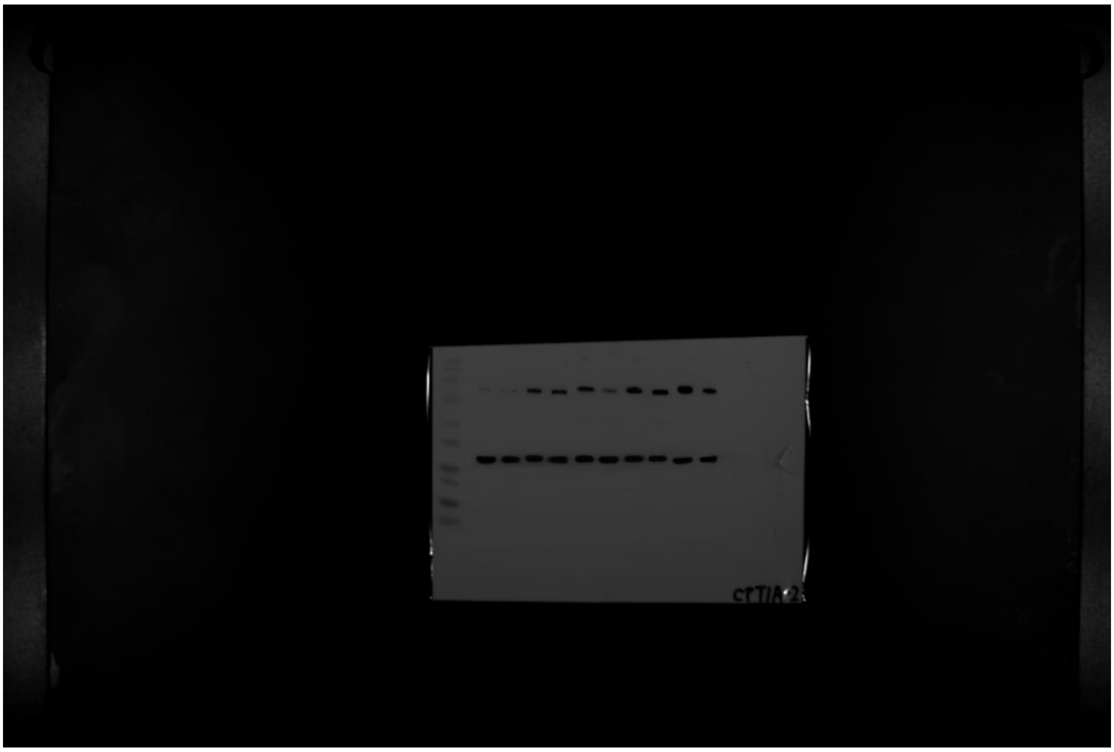

Figure 1B CPT1A&GAPDH

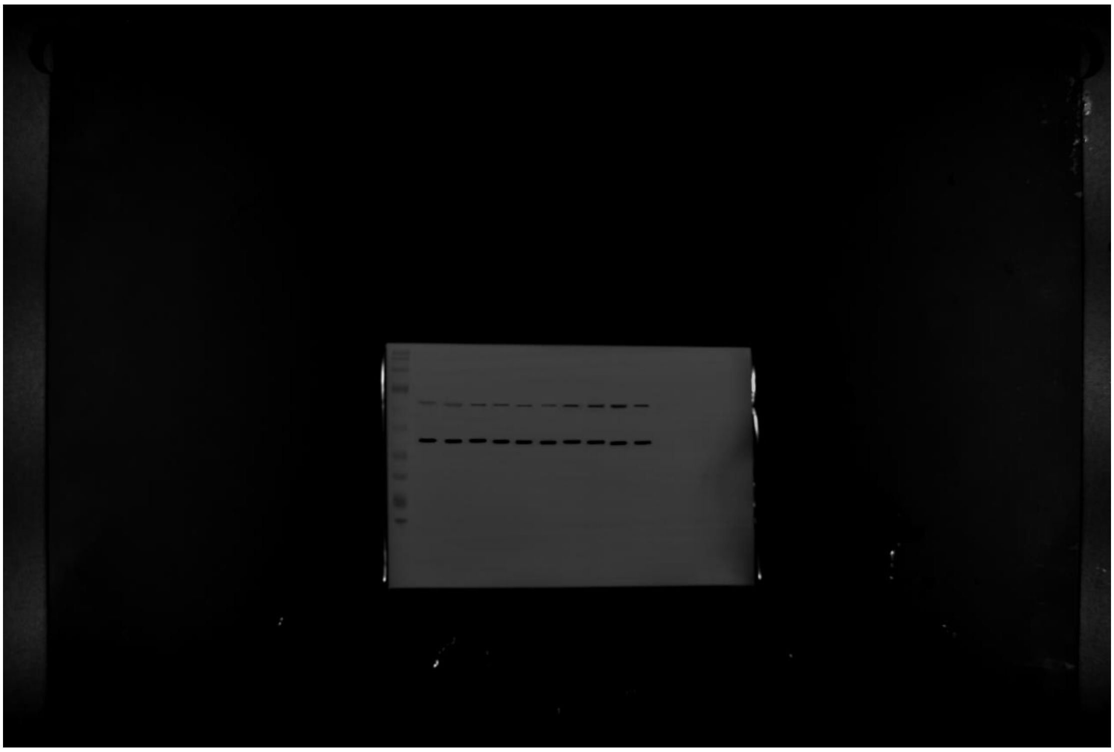

Figure 1B VROT

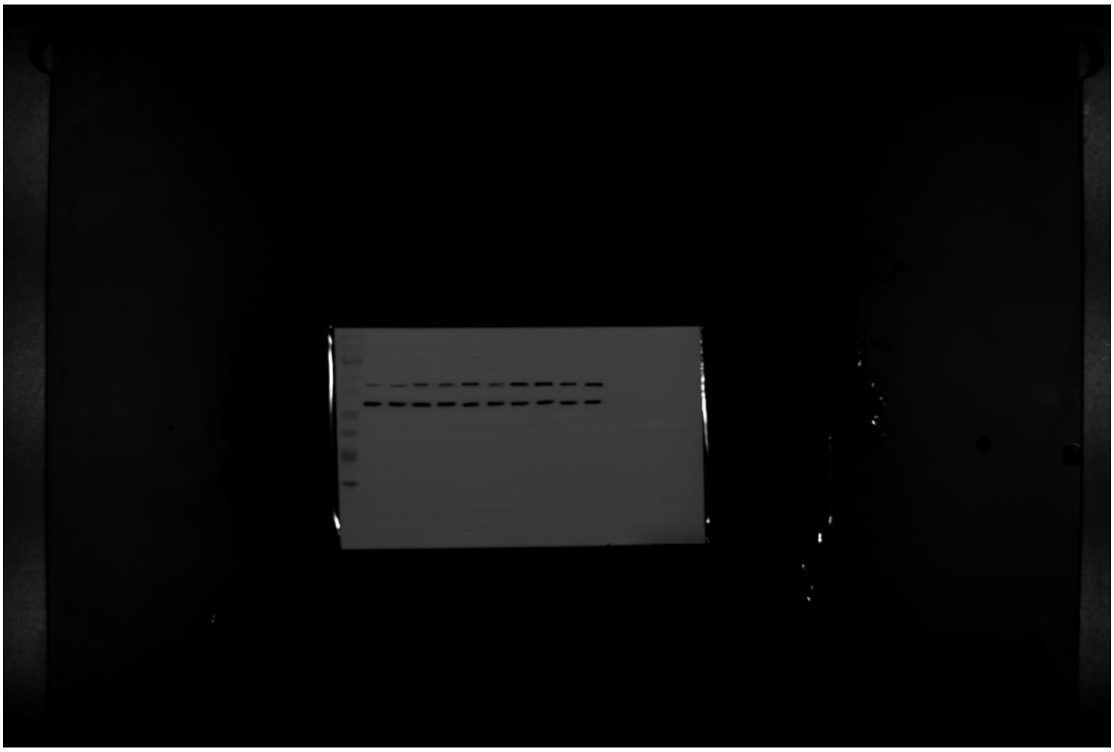

Figure 1B ACADM

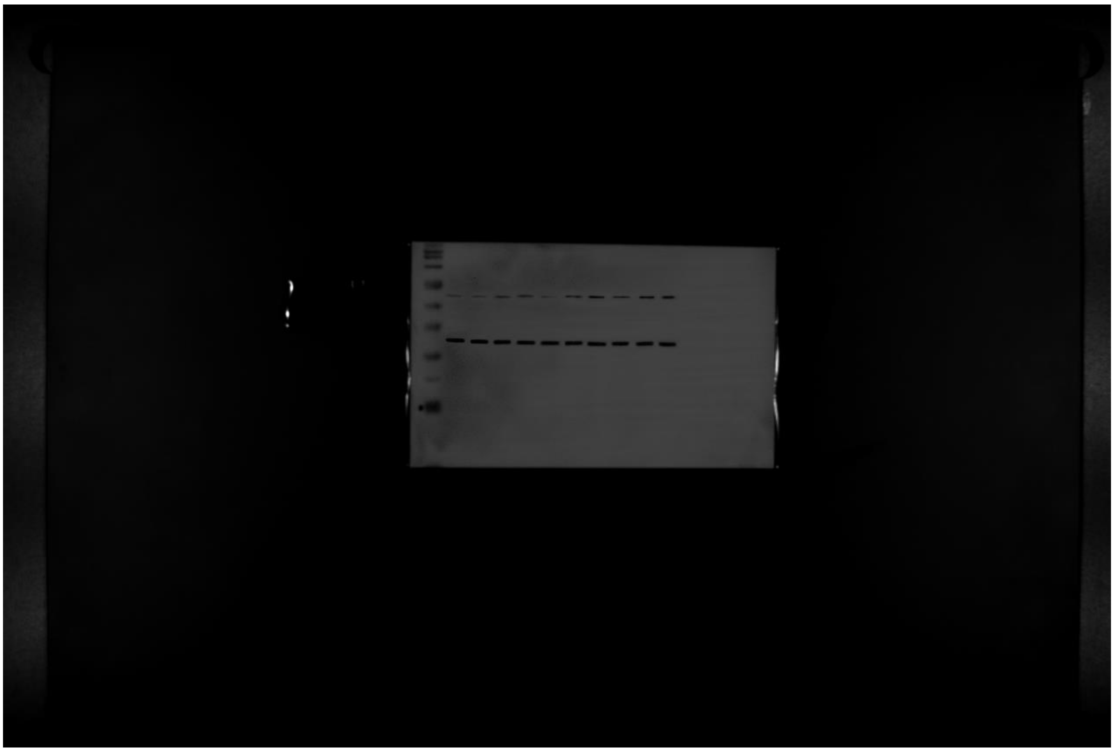

Figure 1B ACSL4

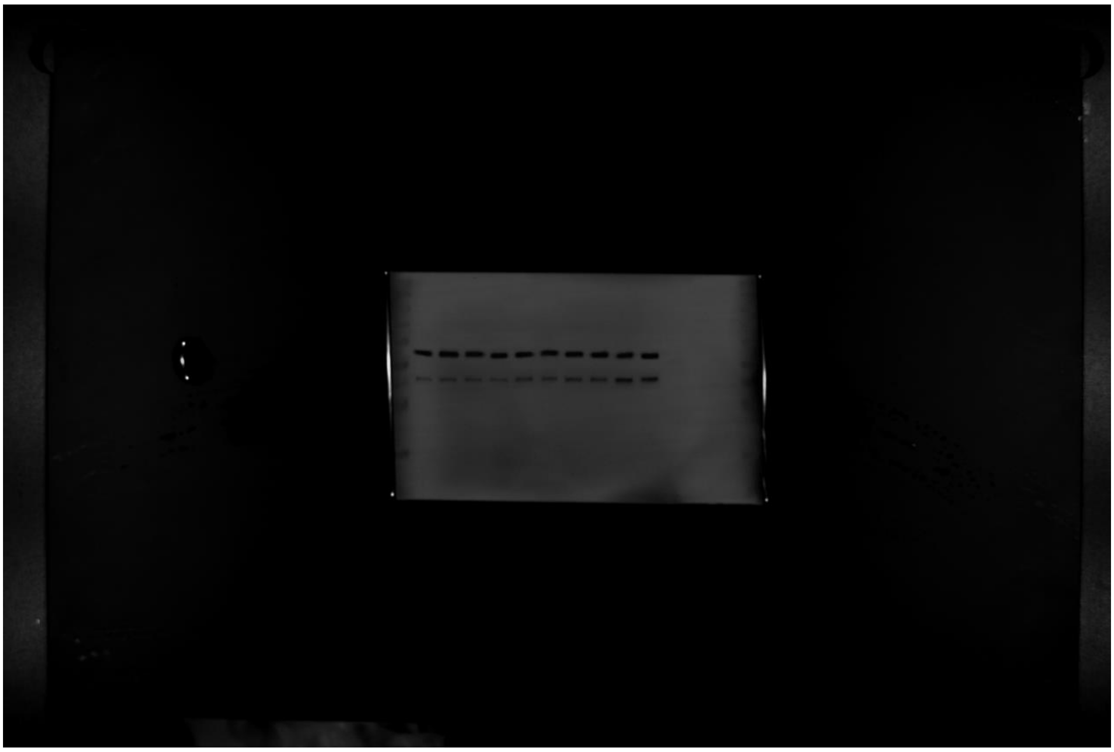

Figure 1B ACOX1

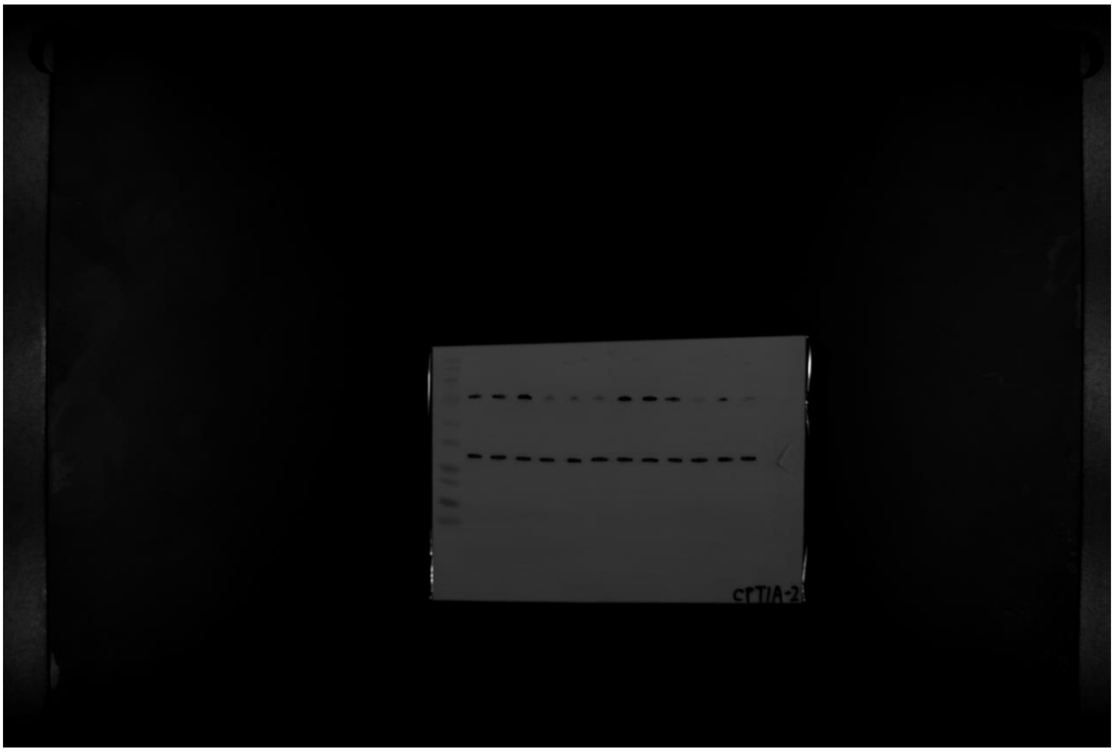

Figure 2A CPT1A&GAPDH

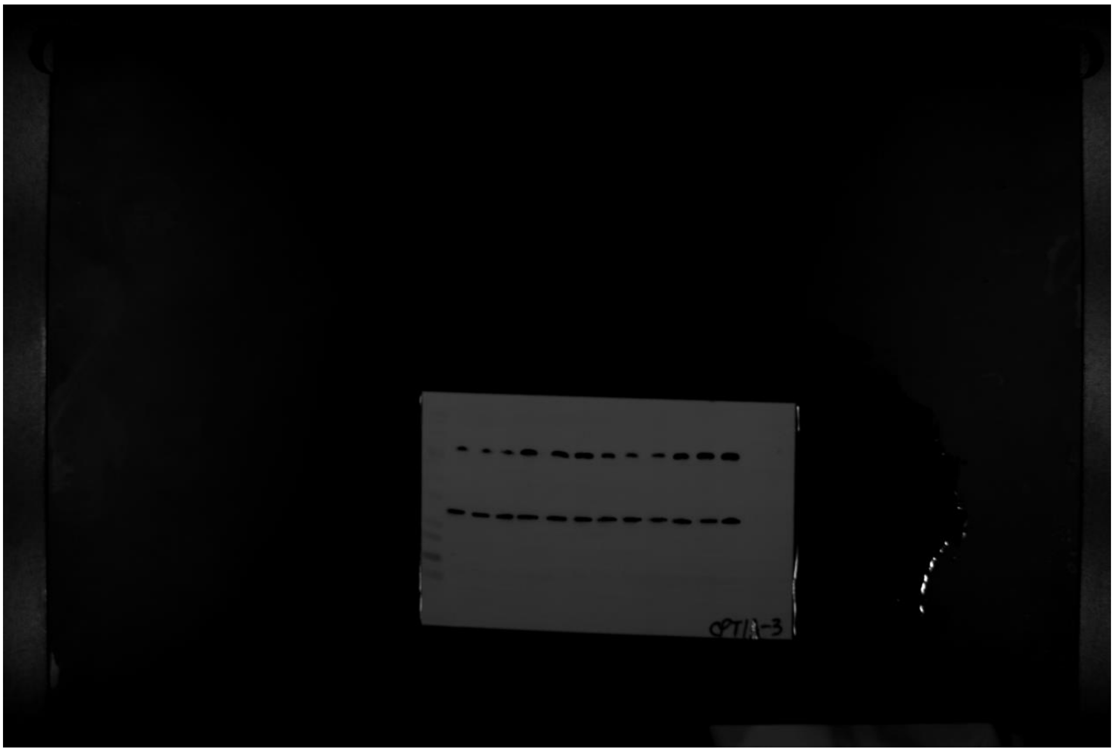

Figure 2A CPT1A&GAPDH

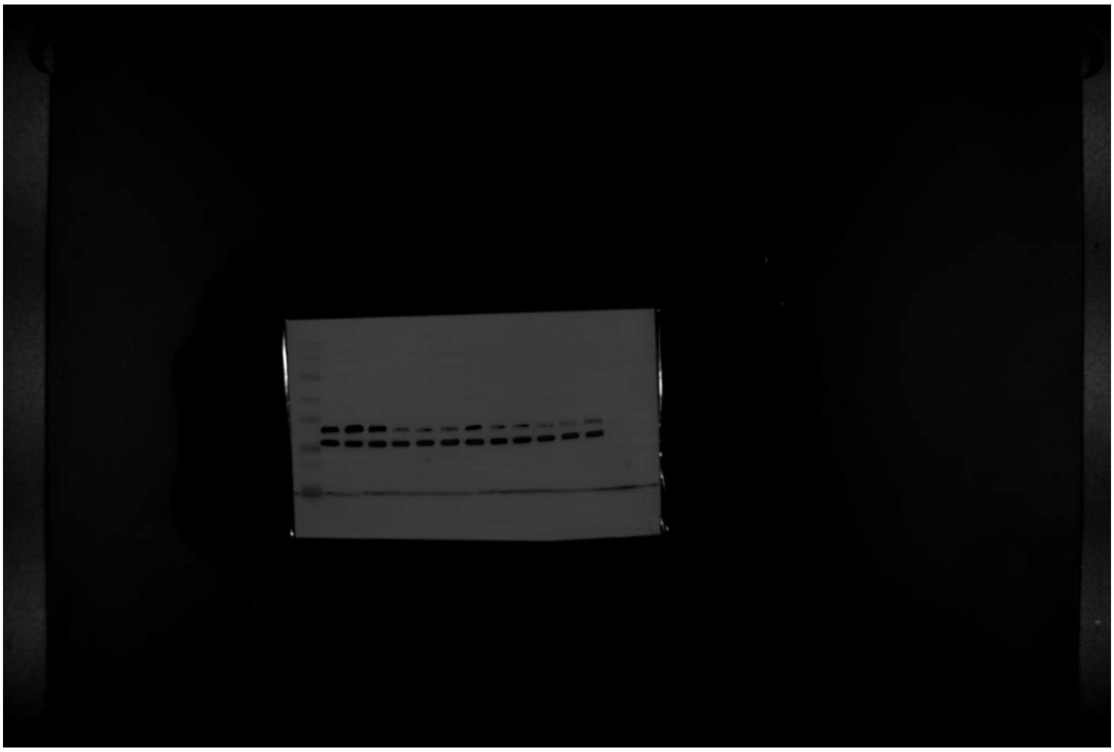

Figure 4B MFF

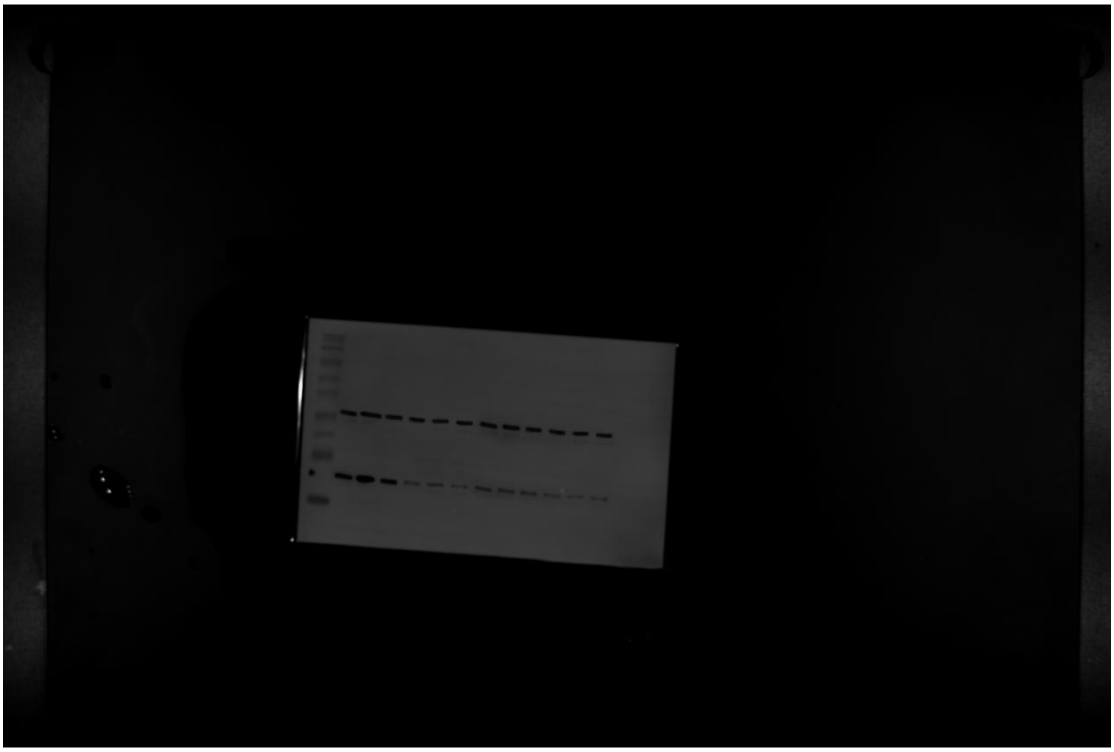

Figure 4B DRP1&GAPDH

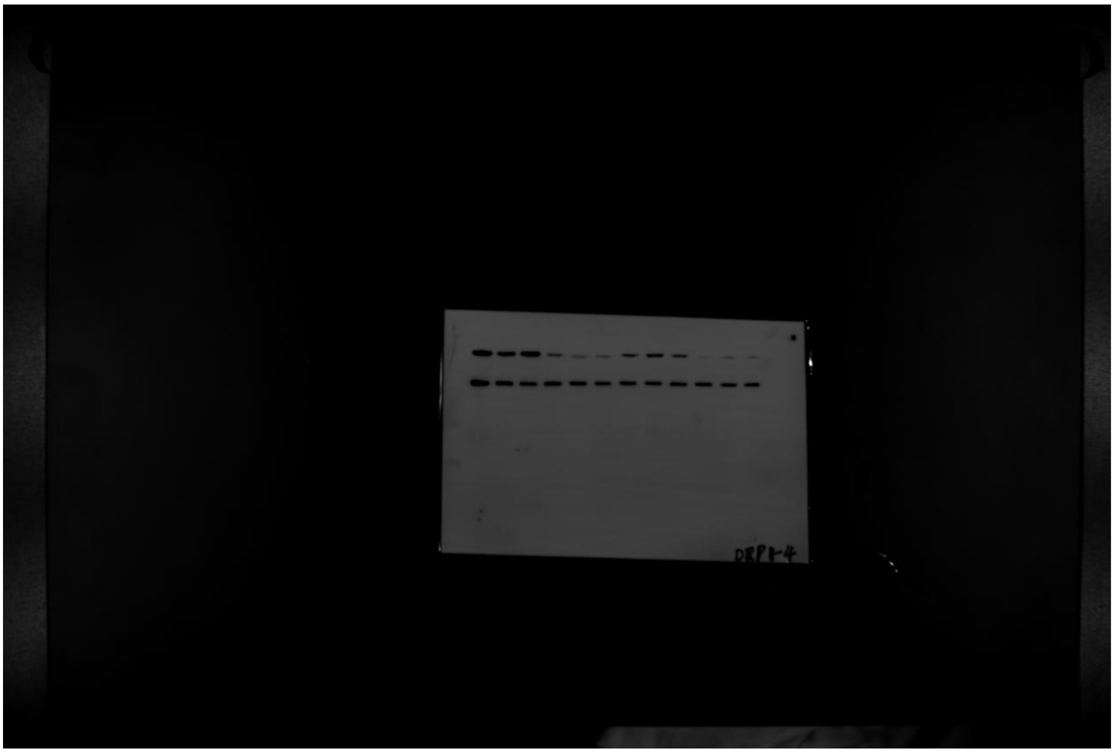

Figure 4B Fis1

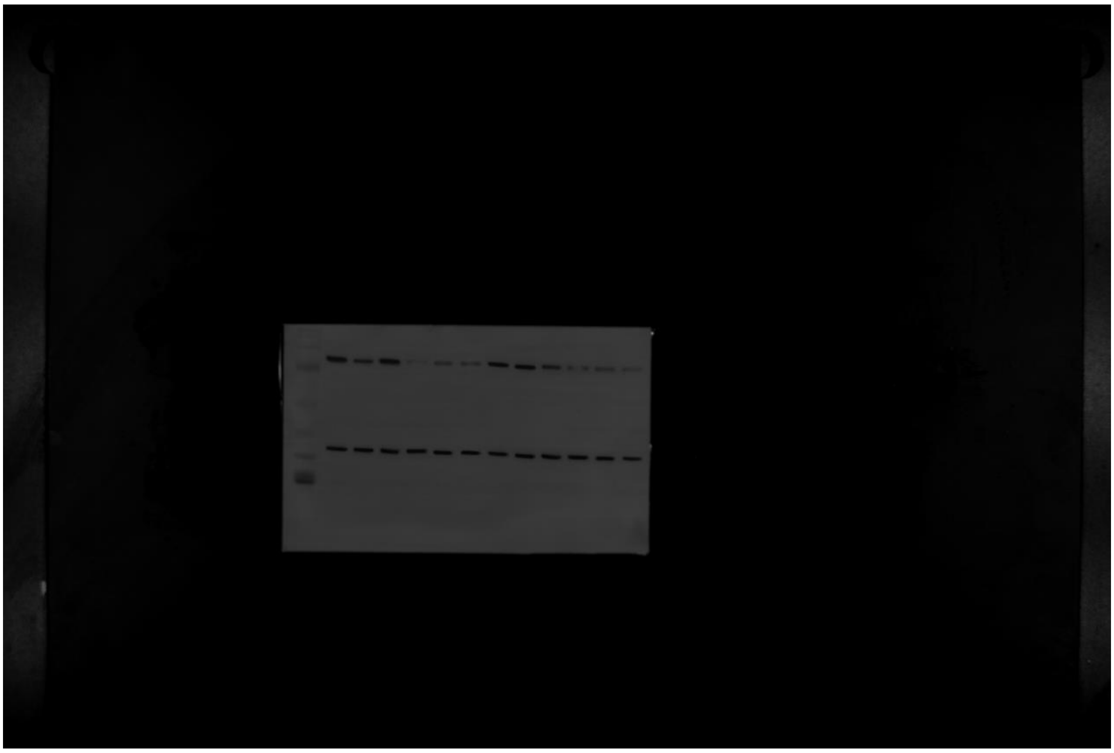

Figure 4B MFN2

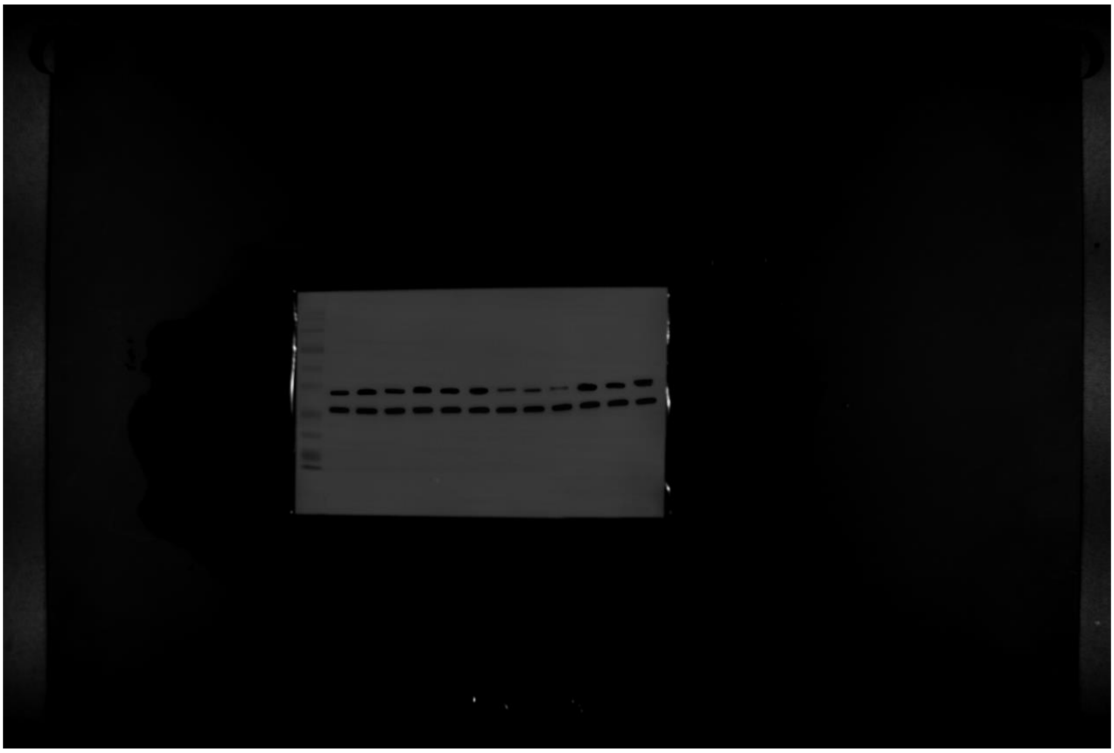

Figure 4B MFF

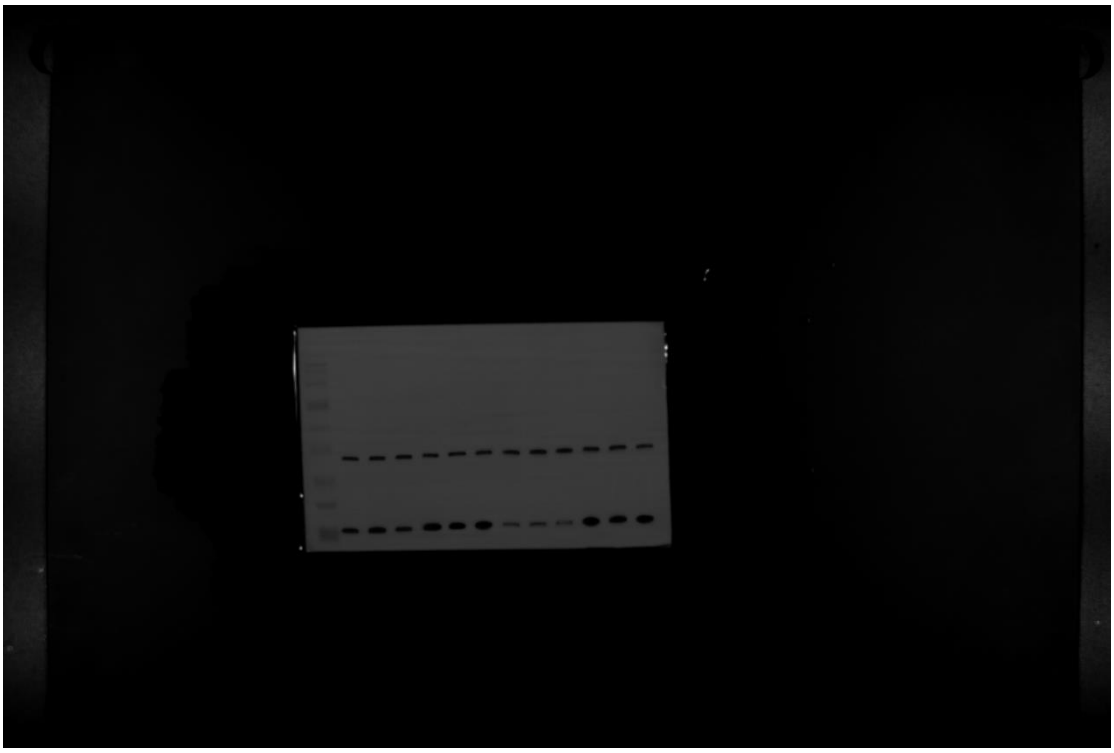

Figure 4B DRP1

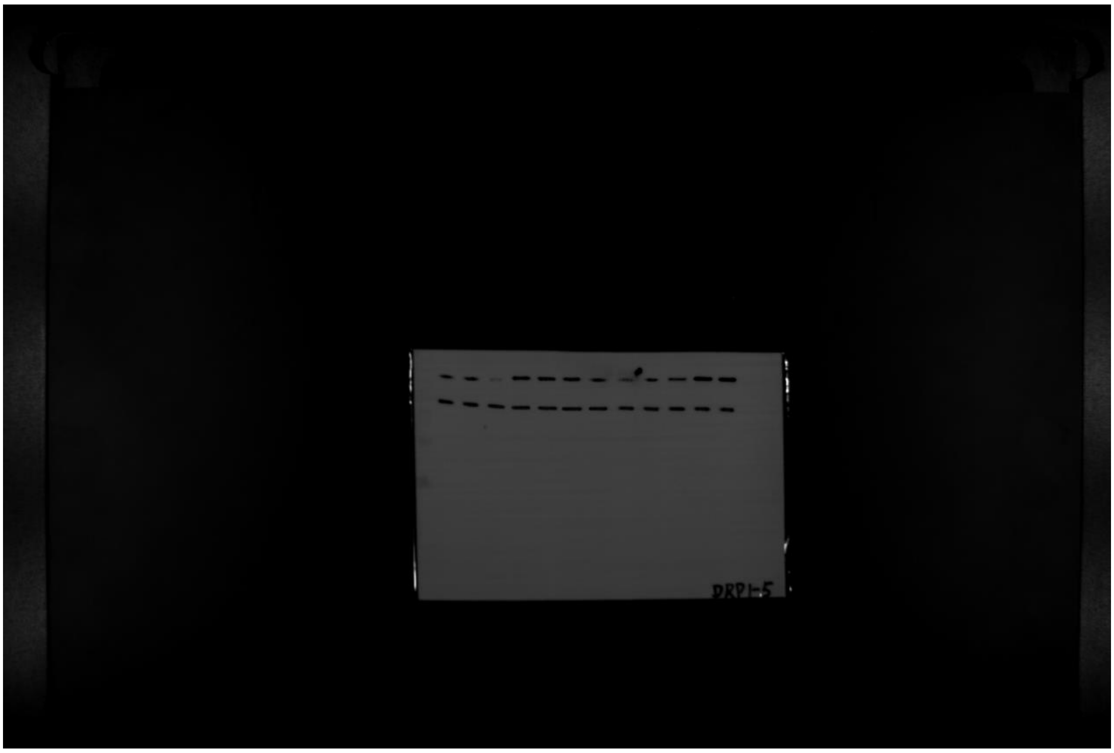

Figure 4B FIS1

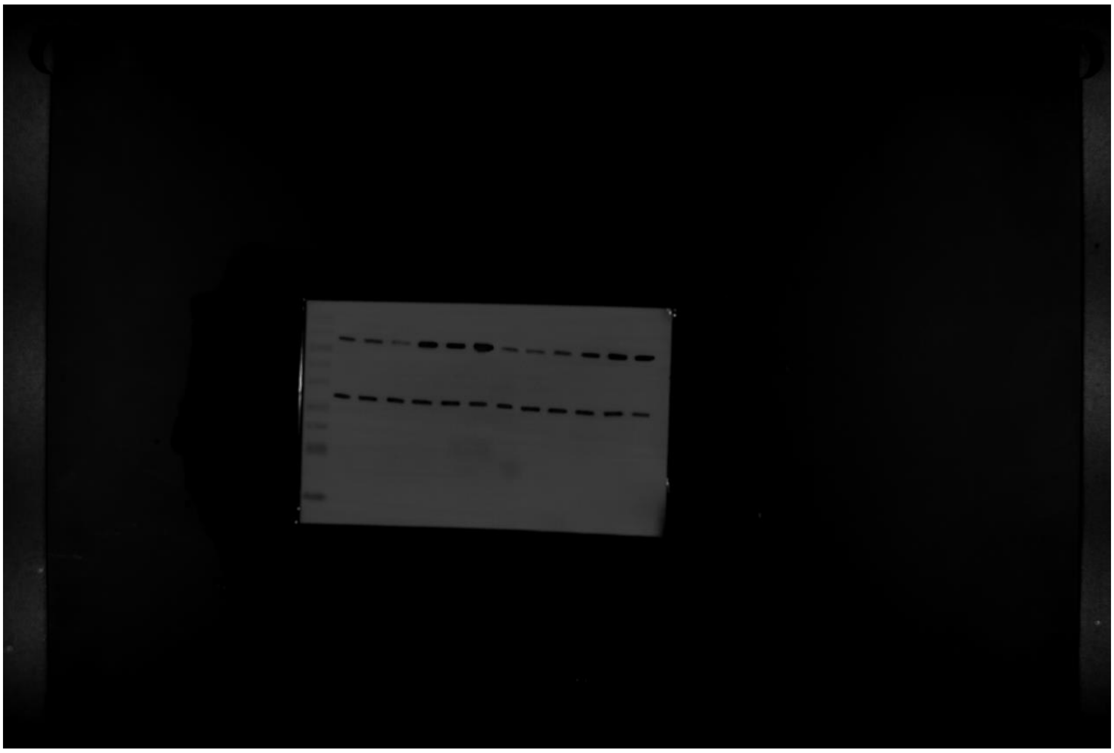

Figure 4B MFN2

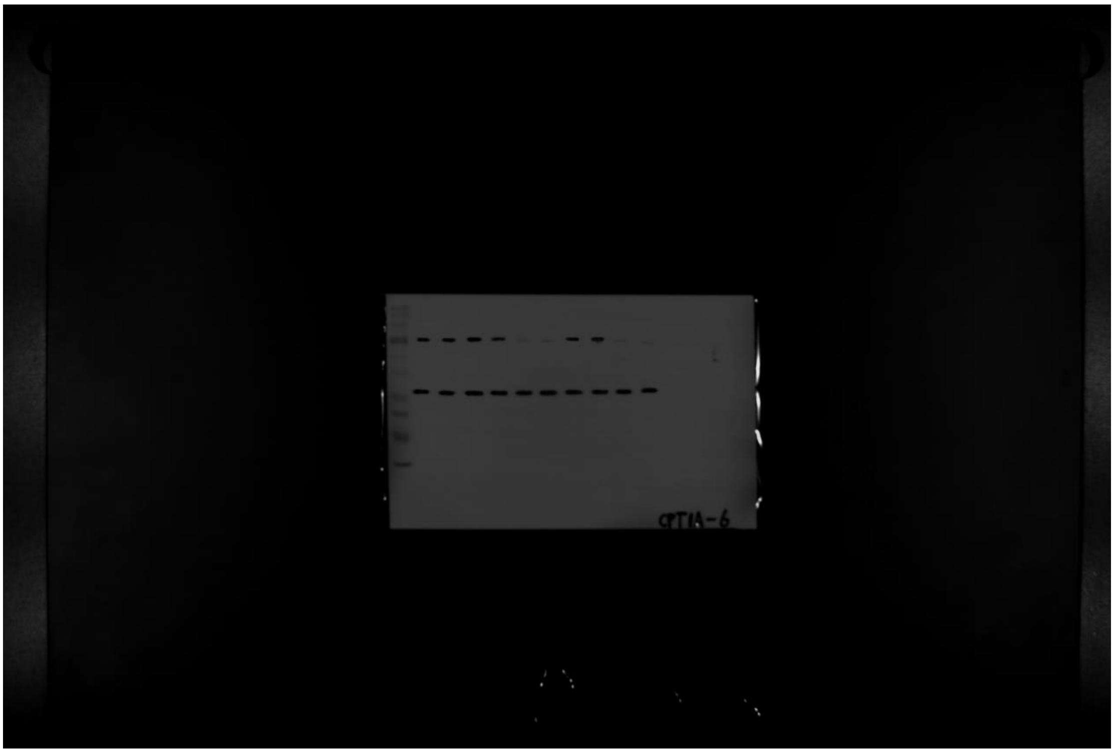

Figure 5B CPT1A&GAPDH

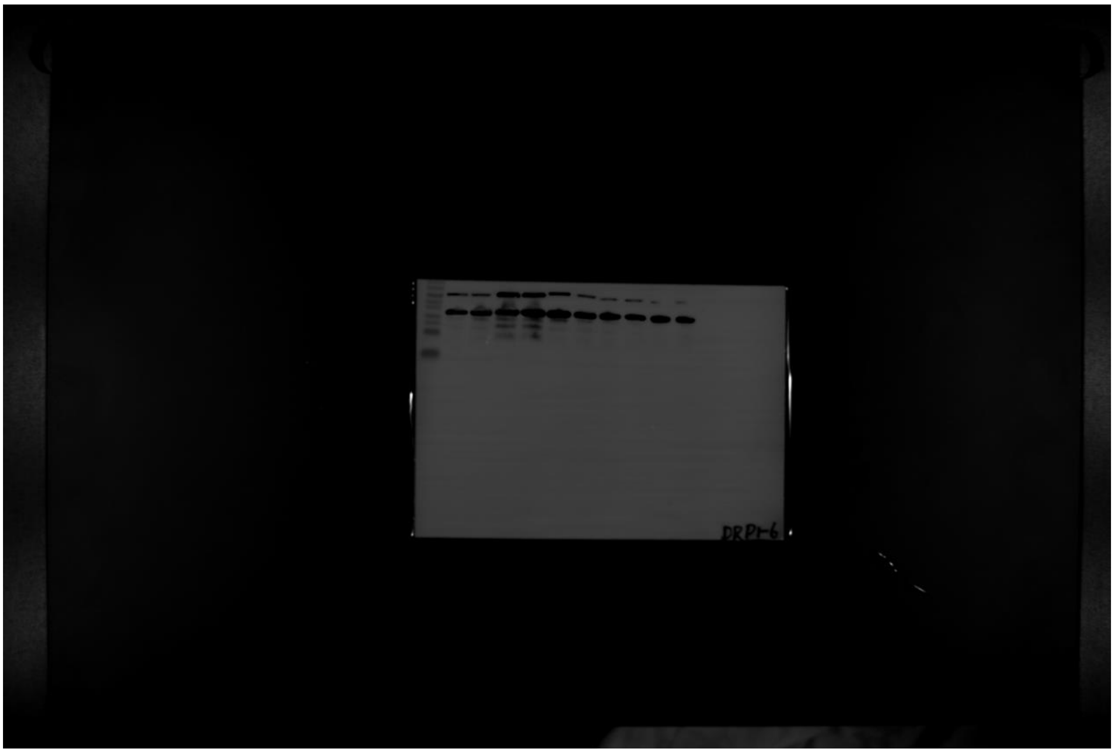

Figure 5B DRP1

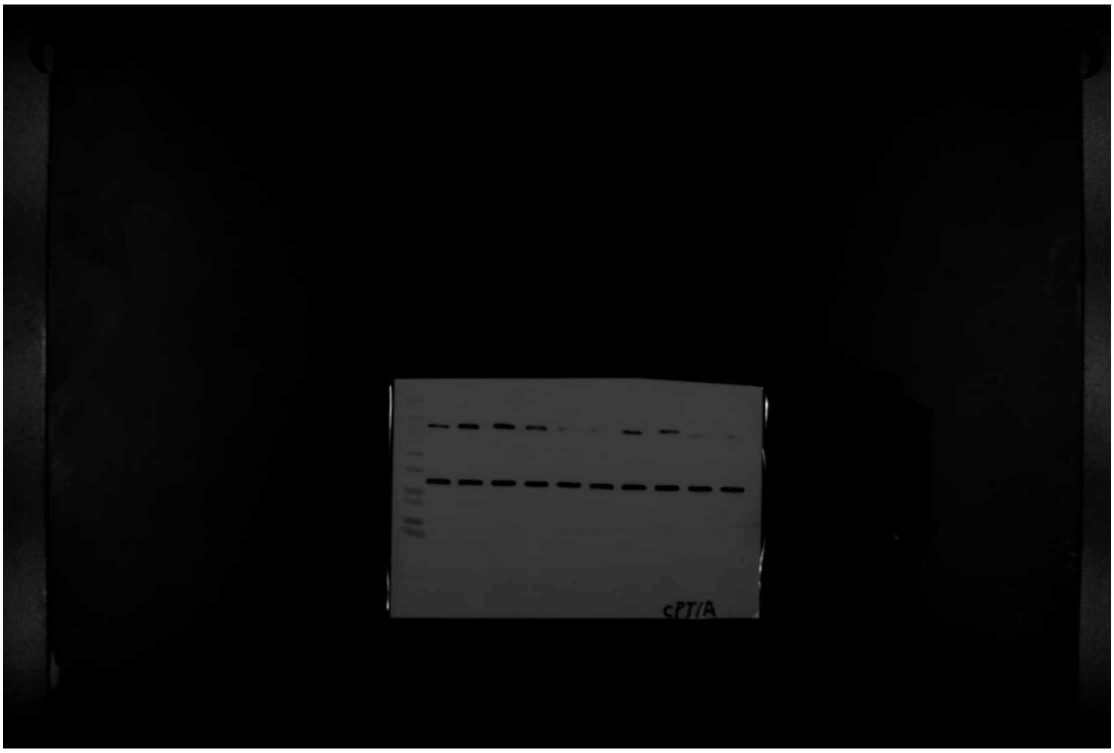

Figure 5B CPT1A&GAPDH

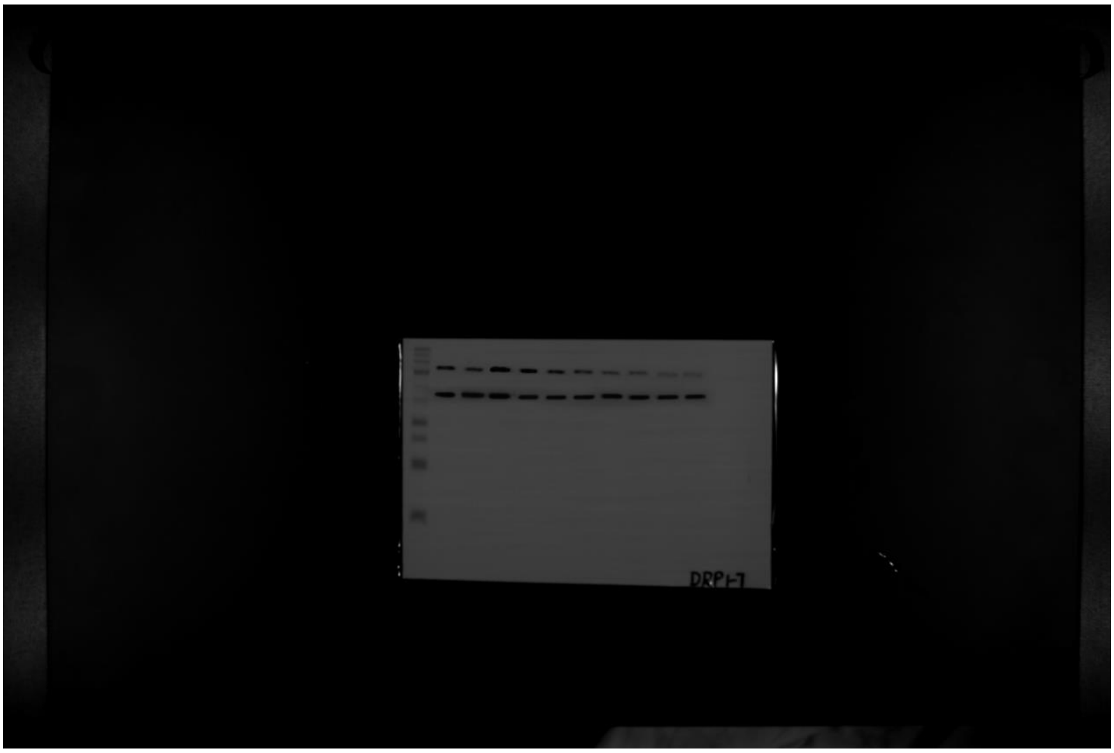

Figure 5B DRP1

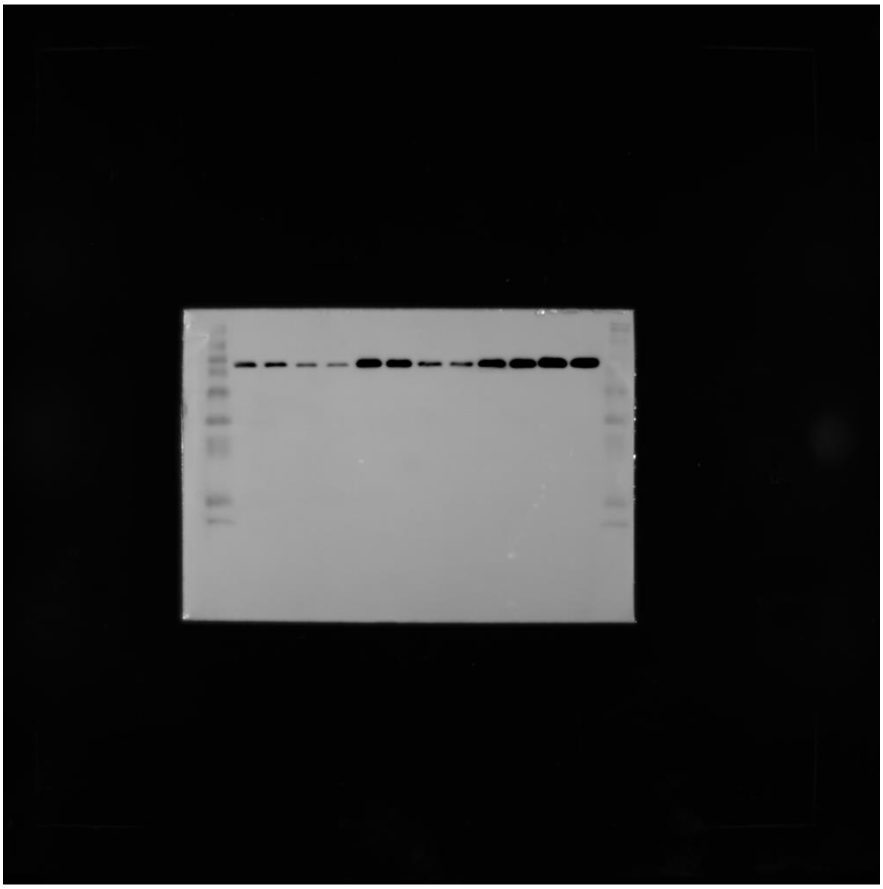

Figure 6B DRP1-Acetyl-lys

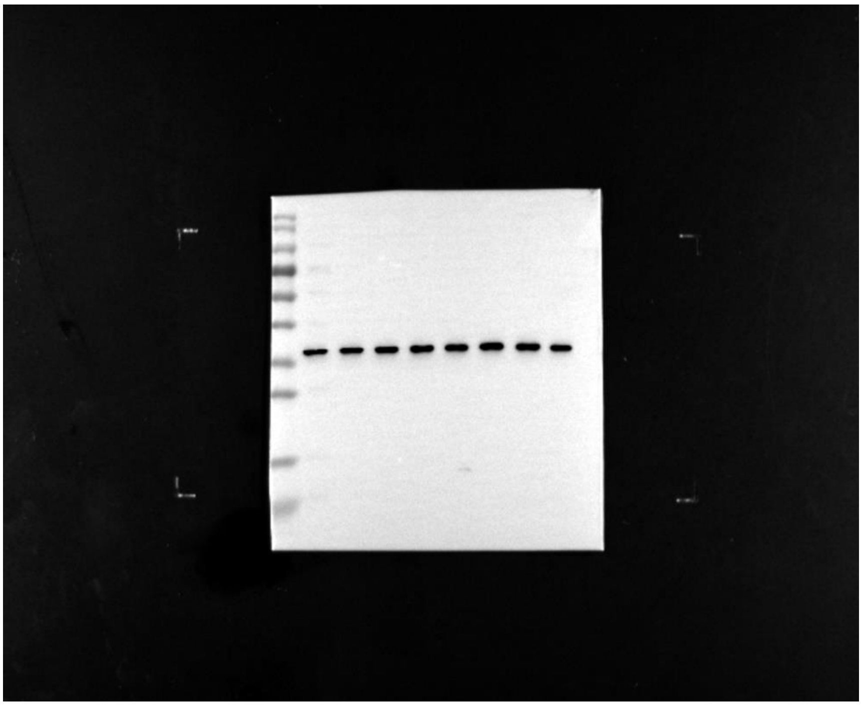

Figure 6B GAPDH

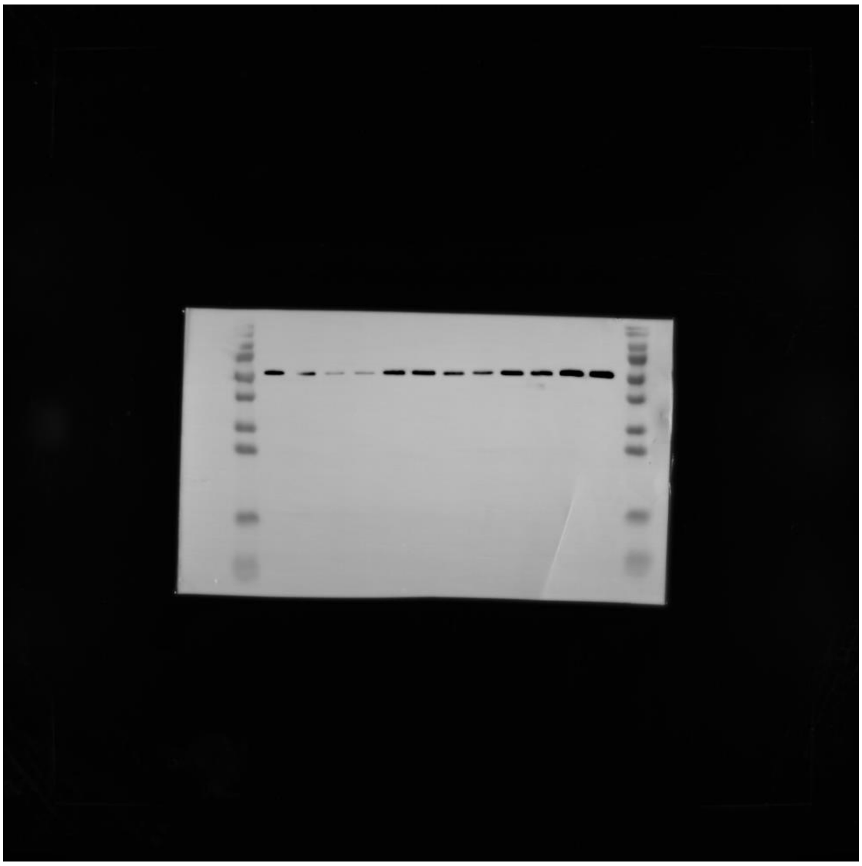

Figure 6B DRP1-Acetyl-lys

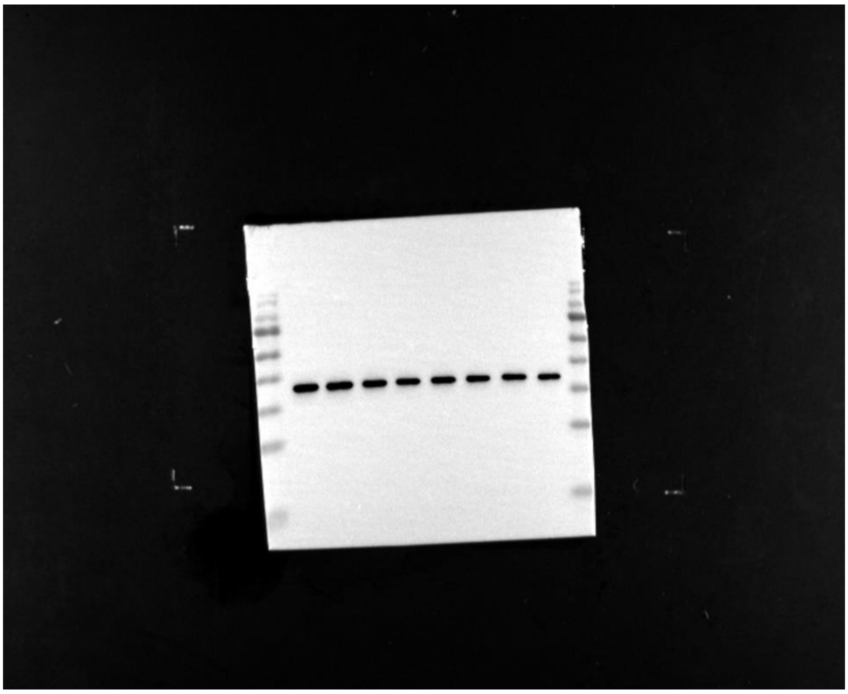

Figure 6B GAPDH

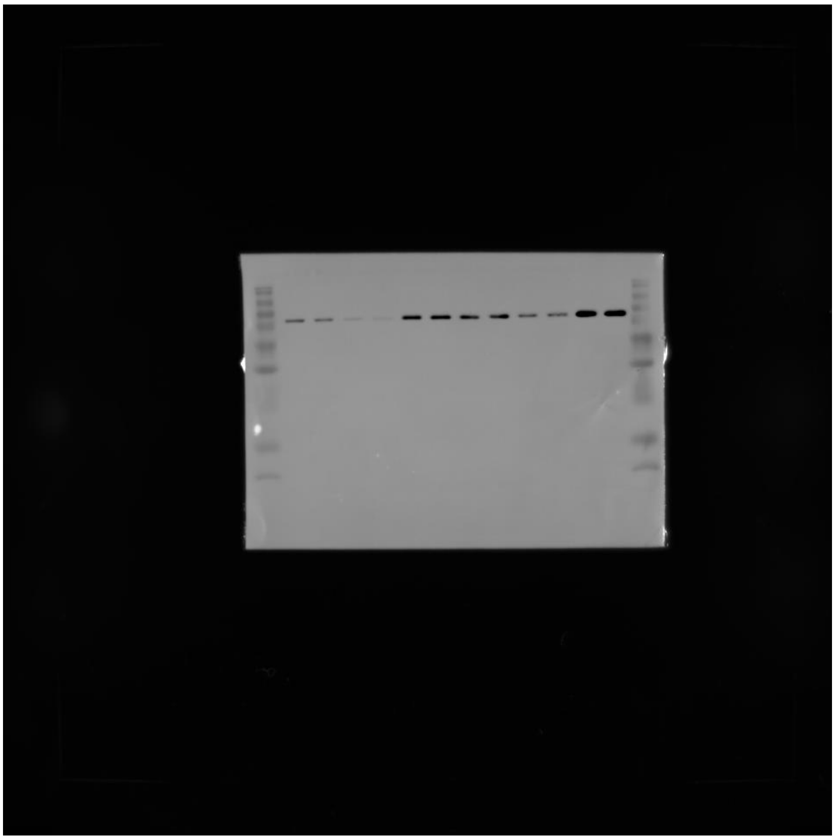

Figure 6C DRP1-Acetyl-lys

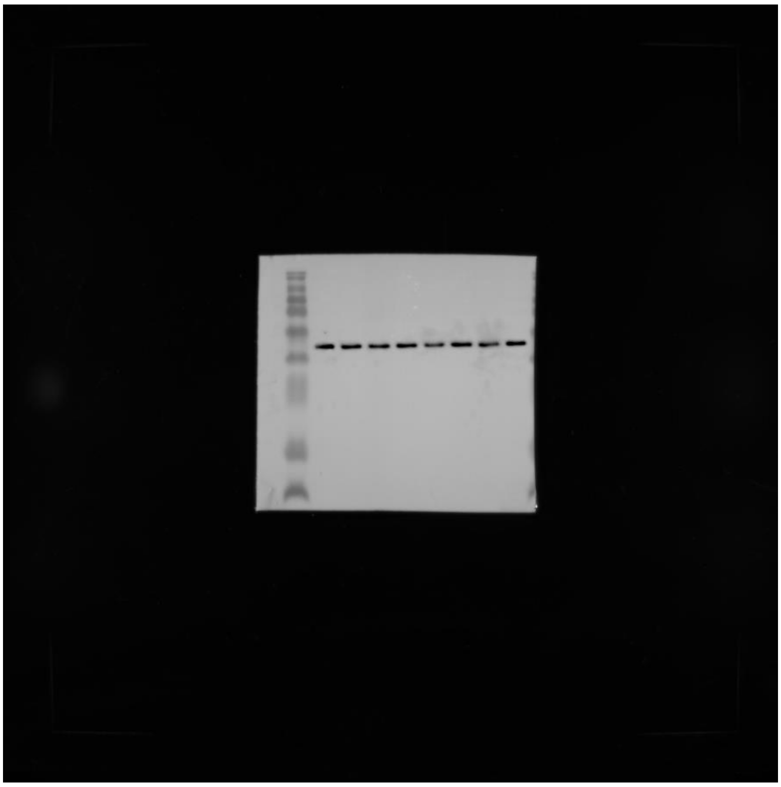

Figure 6C GAPDH

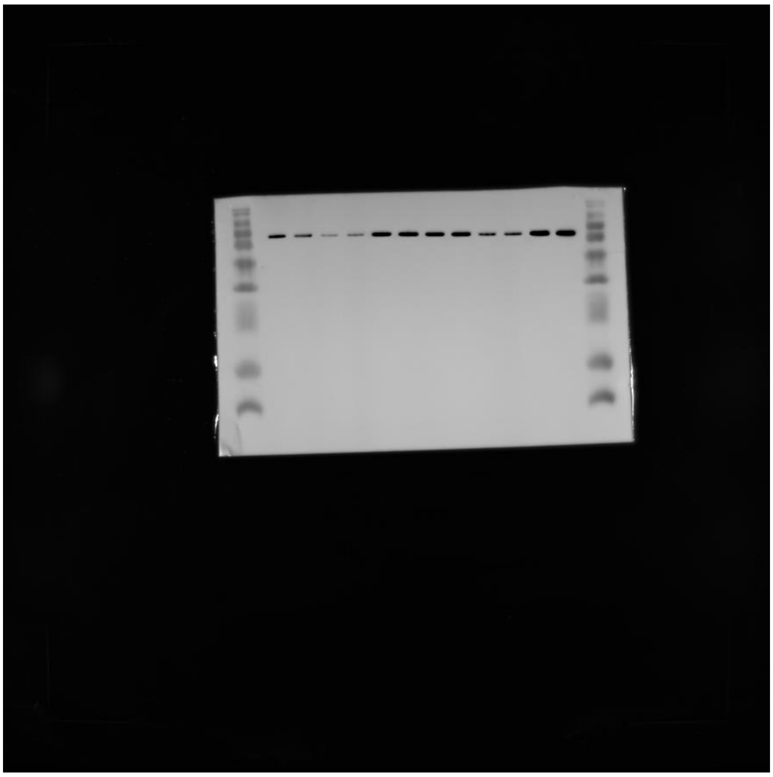

Figure 6C DRP1-Acetyl-lys

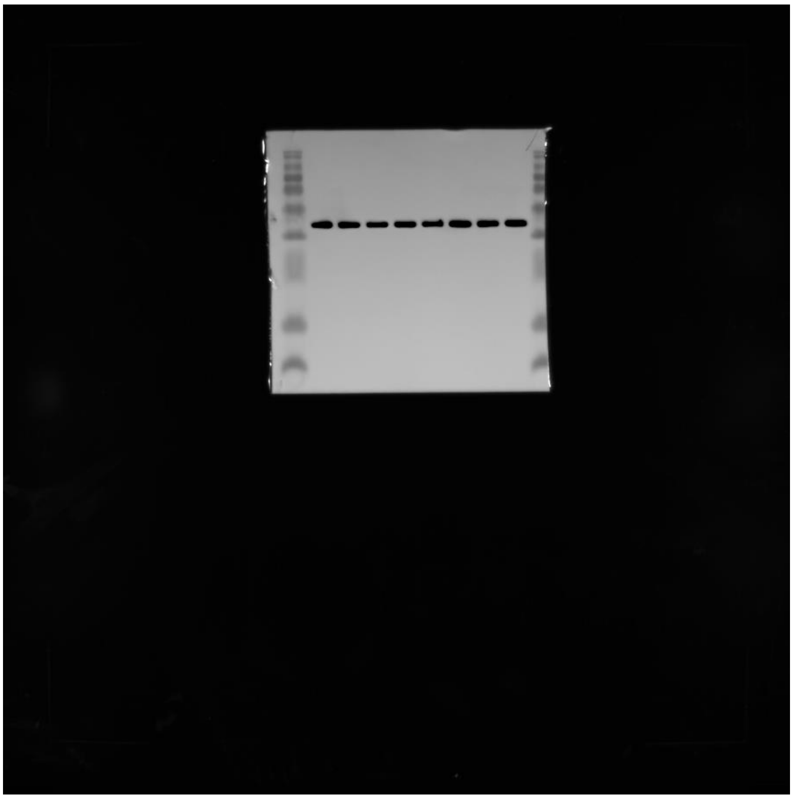

Figure 6C GAPDH

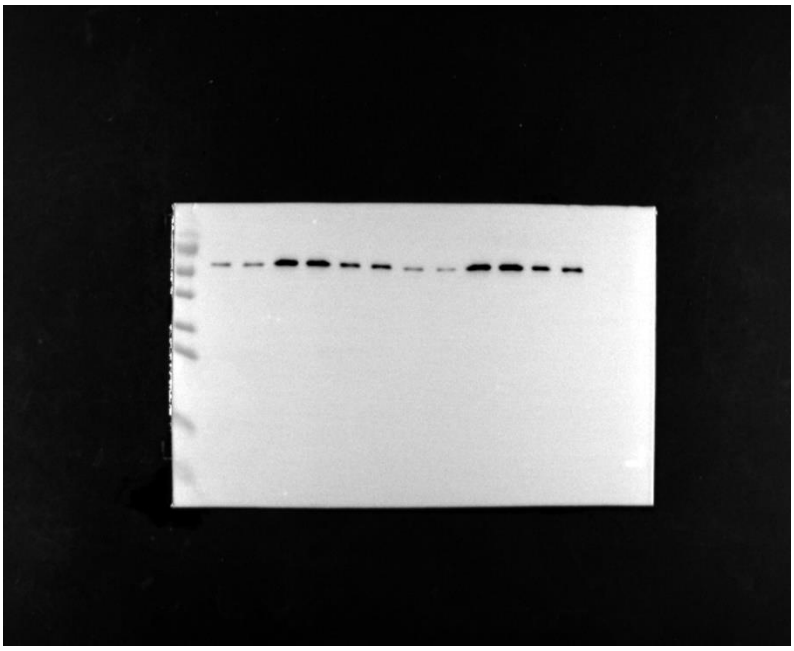

Figure 6D CPT1A

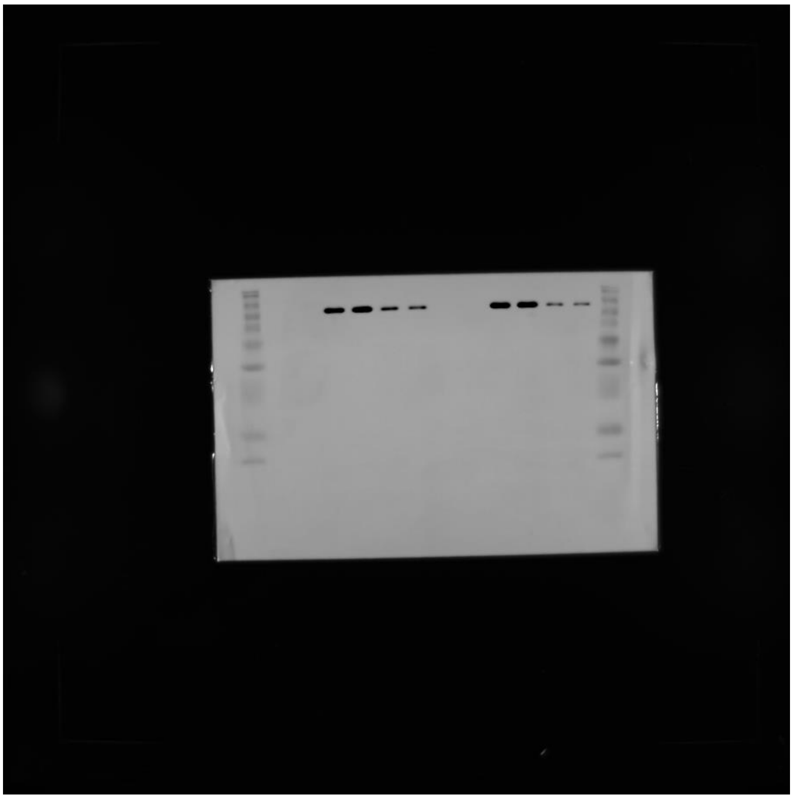

Figure 6D DRP1

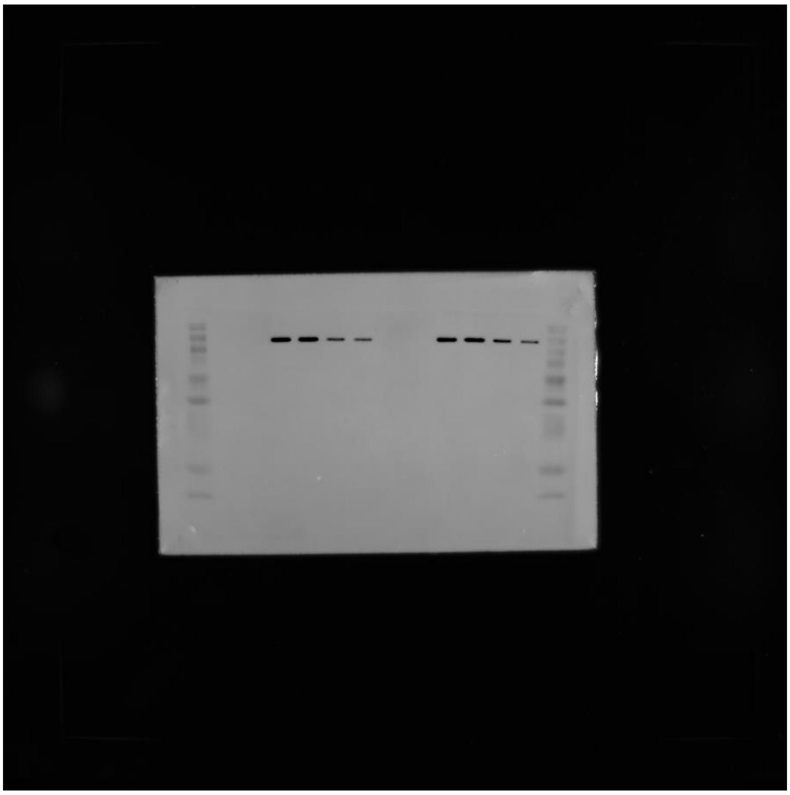

Figure 6D CPT1A

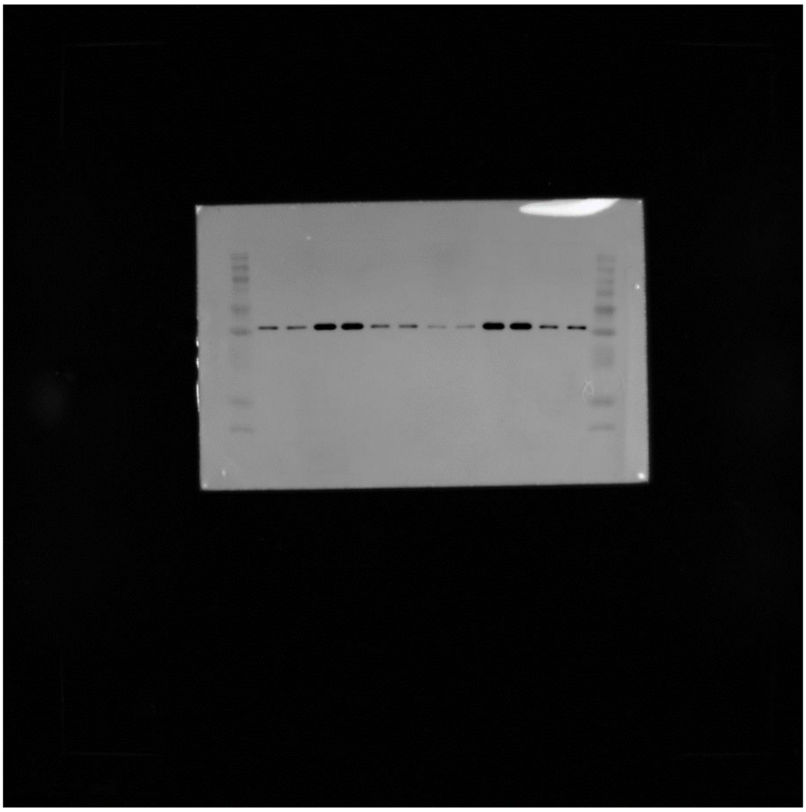

Figure 6D DRP1

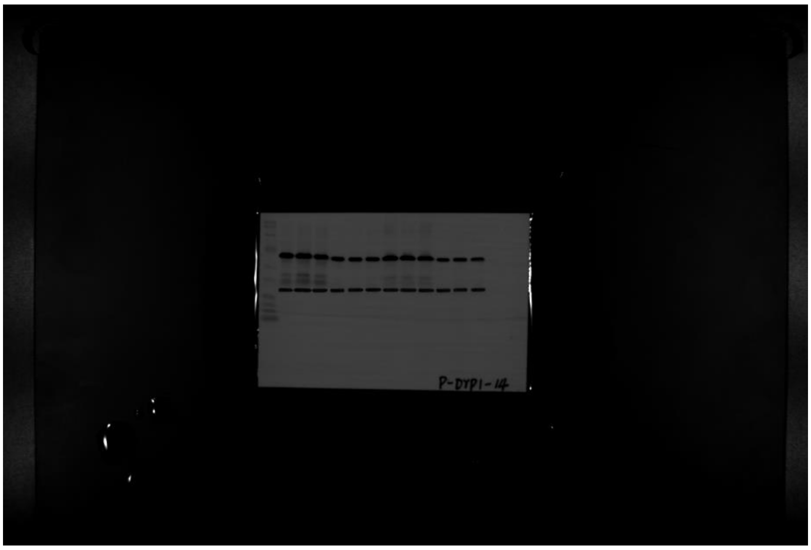

Figure 7A p-DRP1

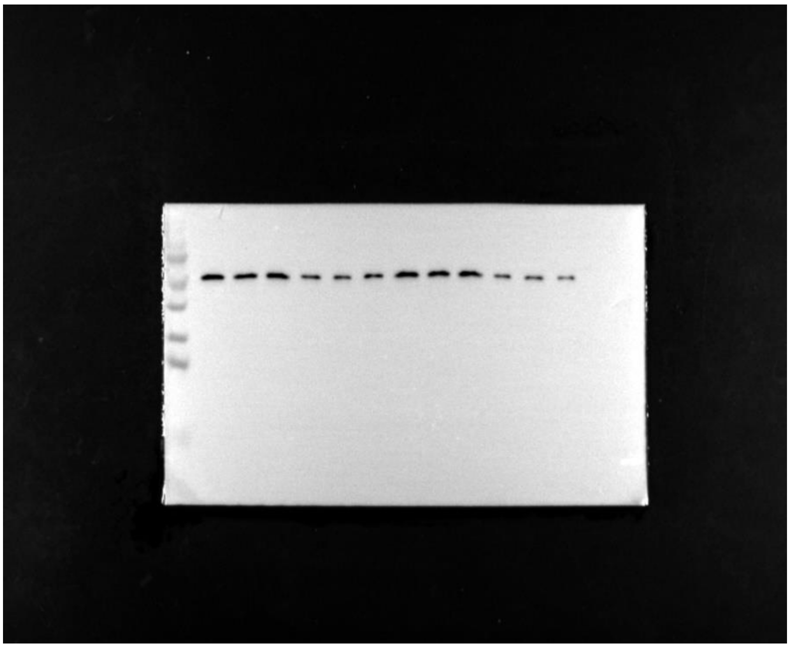

Figure 7A DRP1

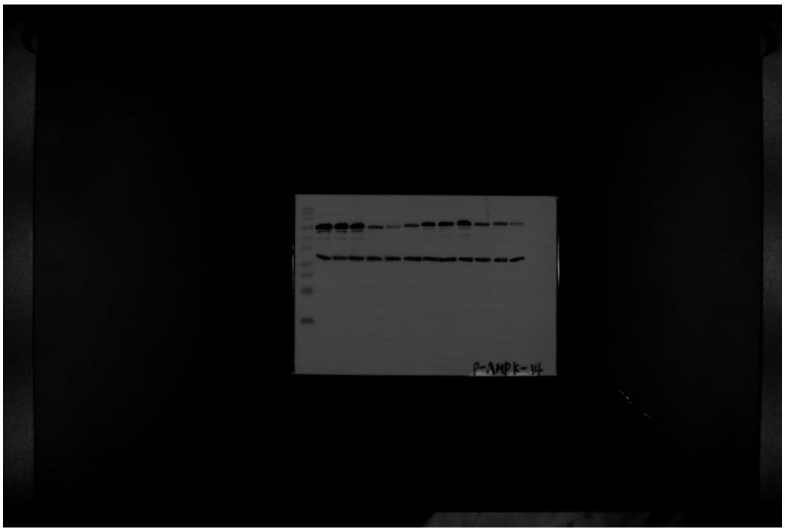

Figure 7A p-AMPK

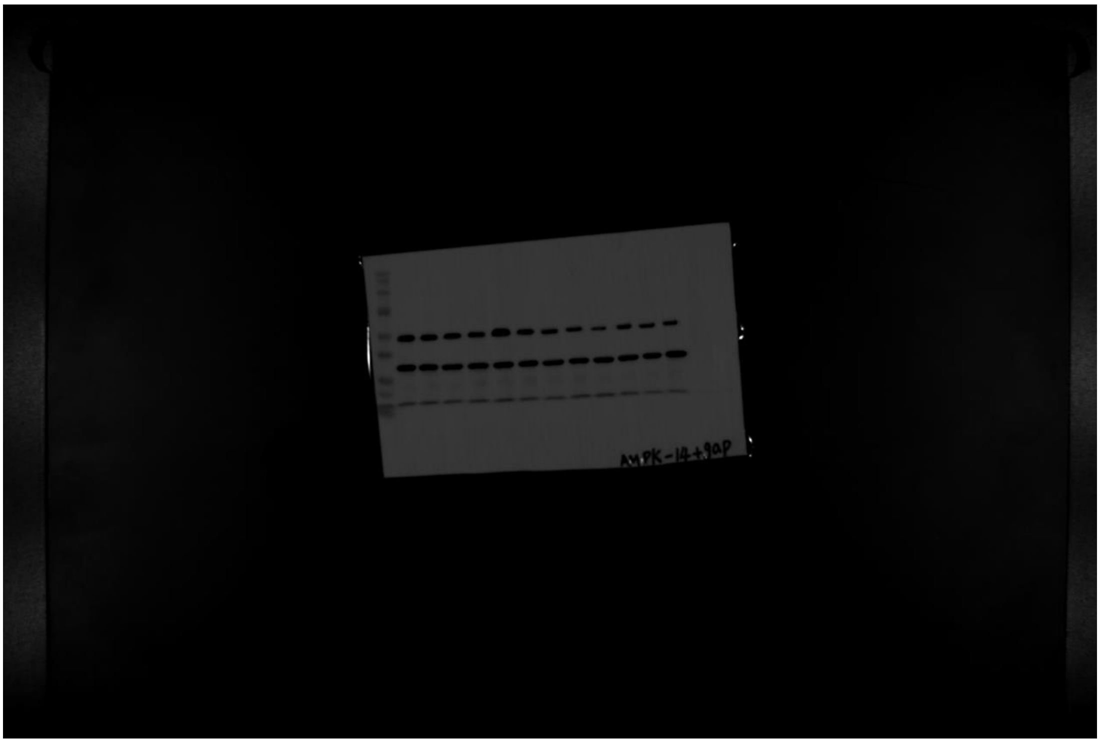

Figure 7A AMPK&GAPDH

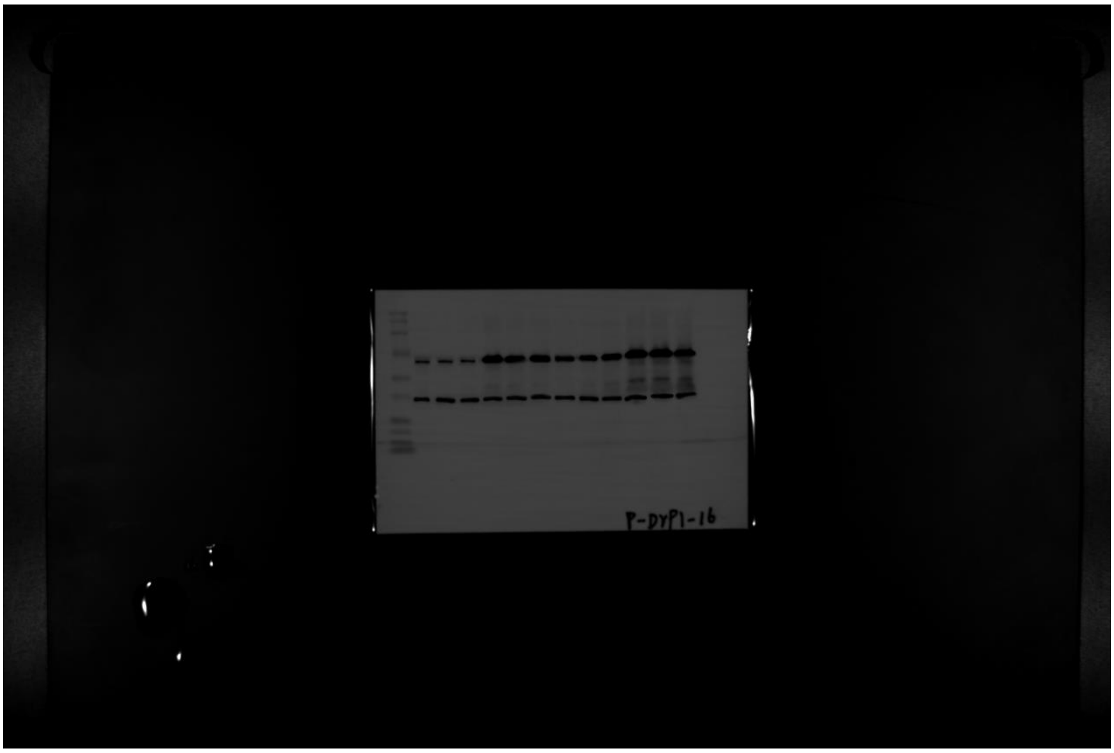

Figure 7A p-DRP1&GAPDH

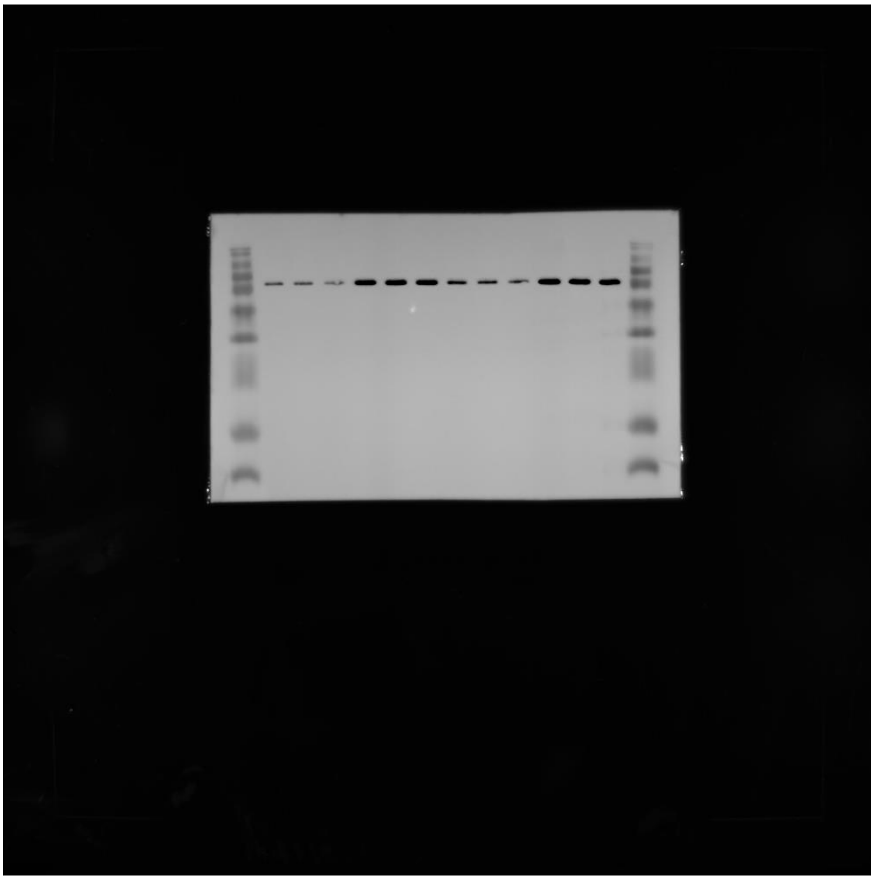

Figure 7A DRP1

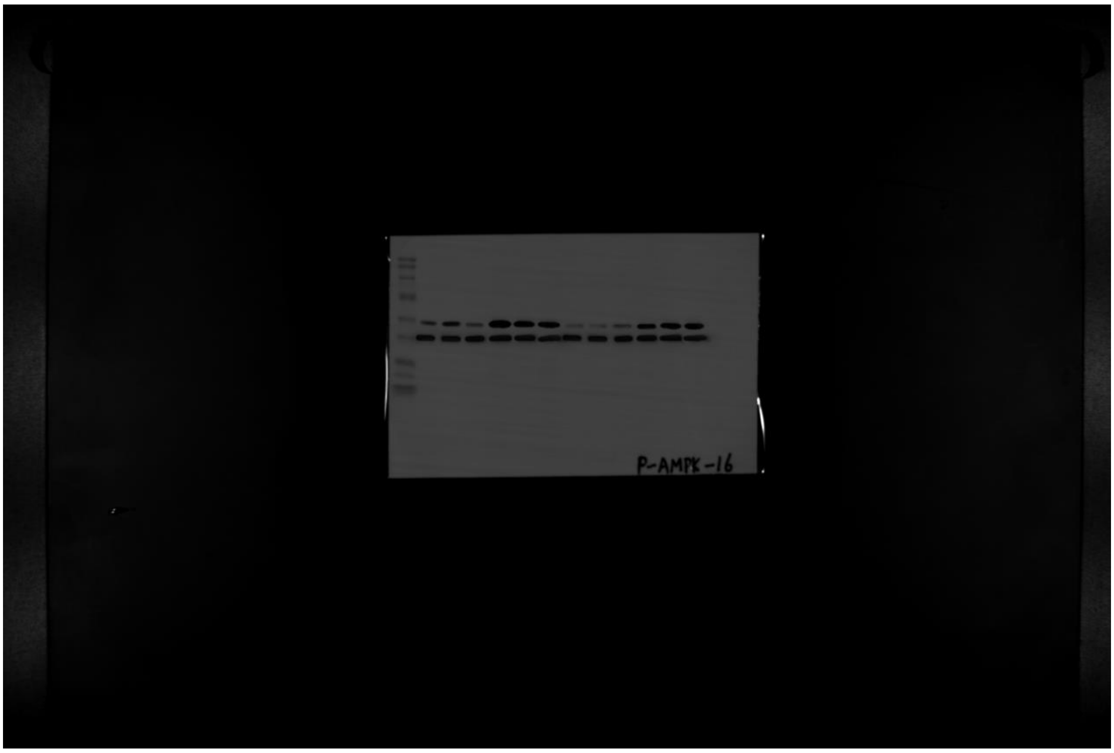

Figure 7A p-AMPK

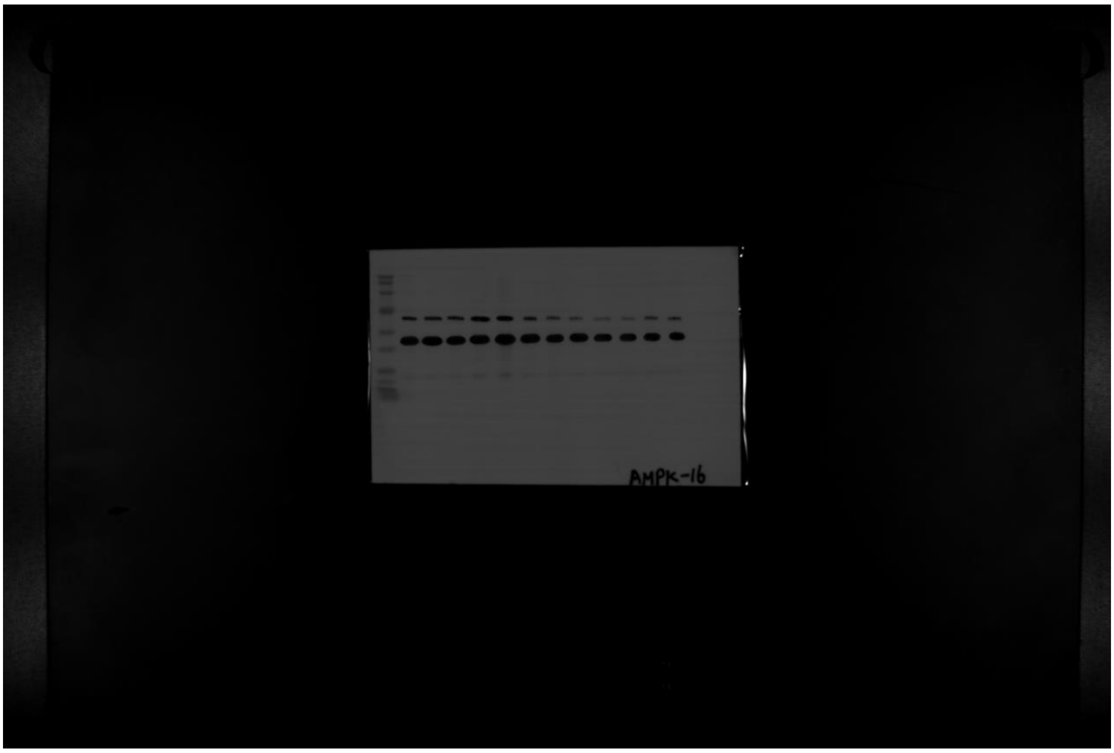

Figure 7A p-AMPK

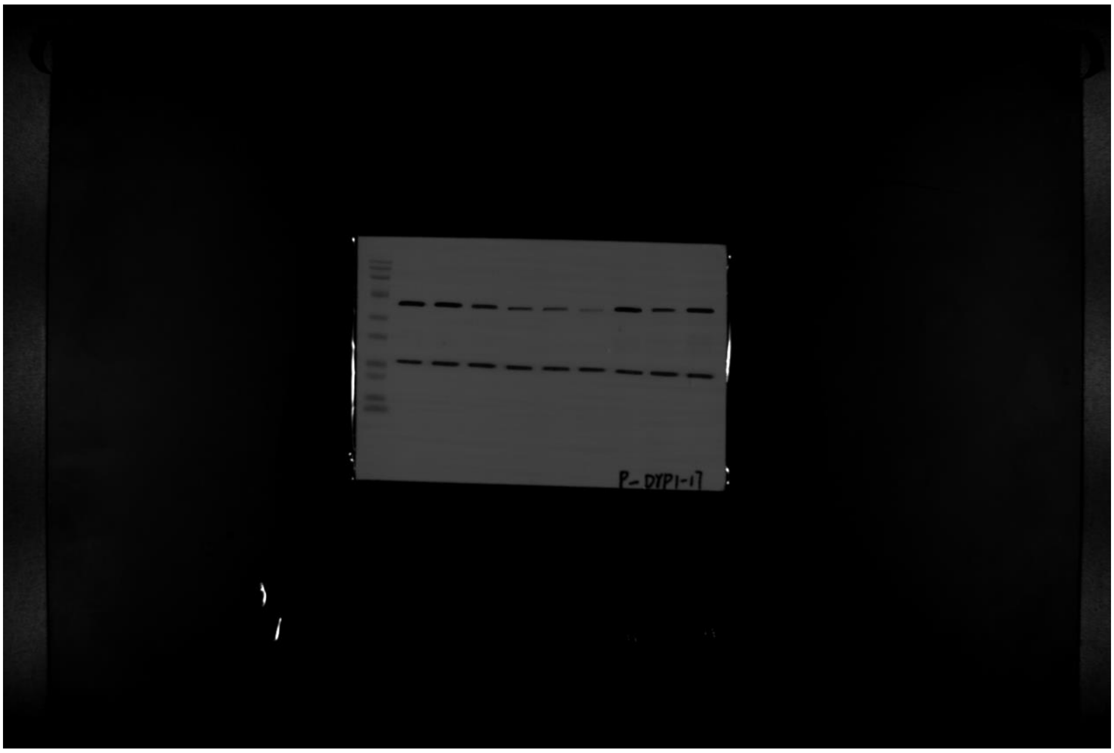

Figure 7B p-DRP1

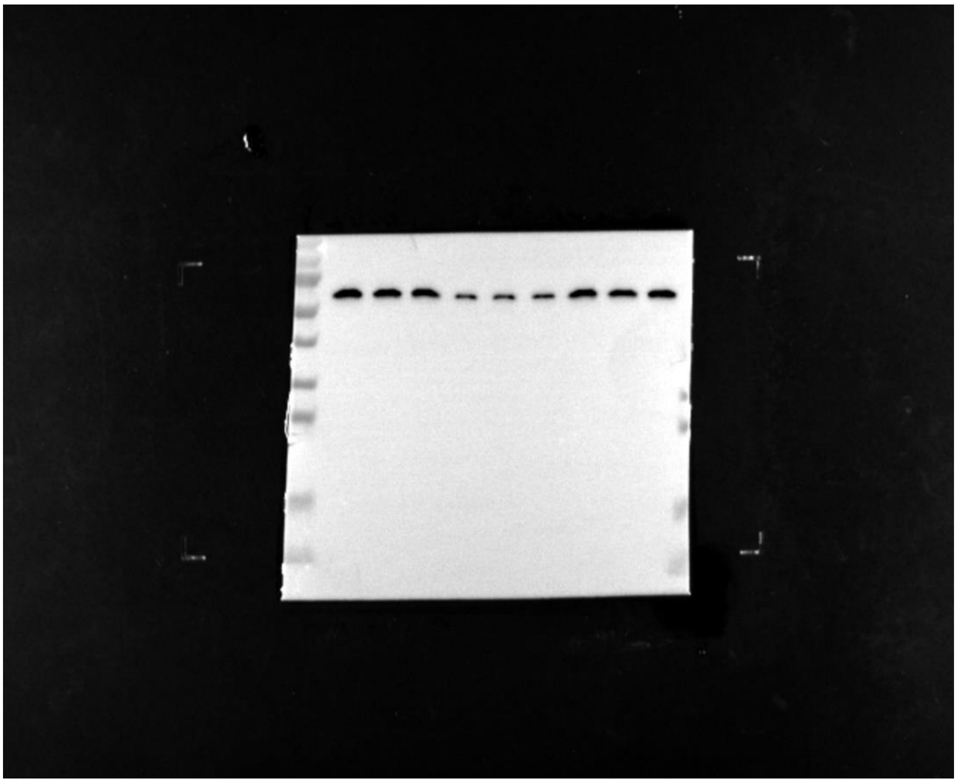

Figure 7B DRP1

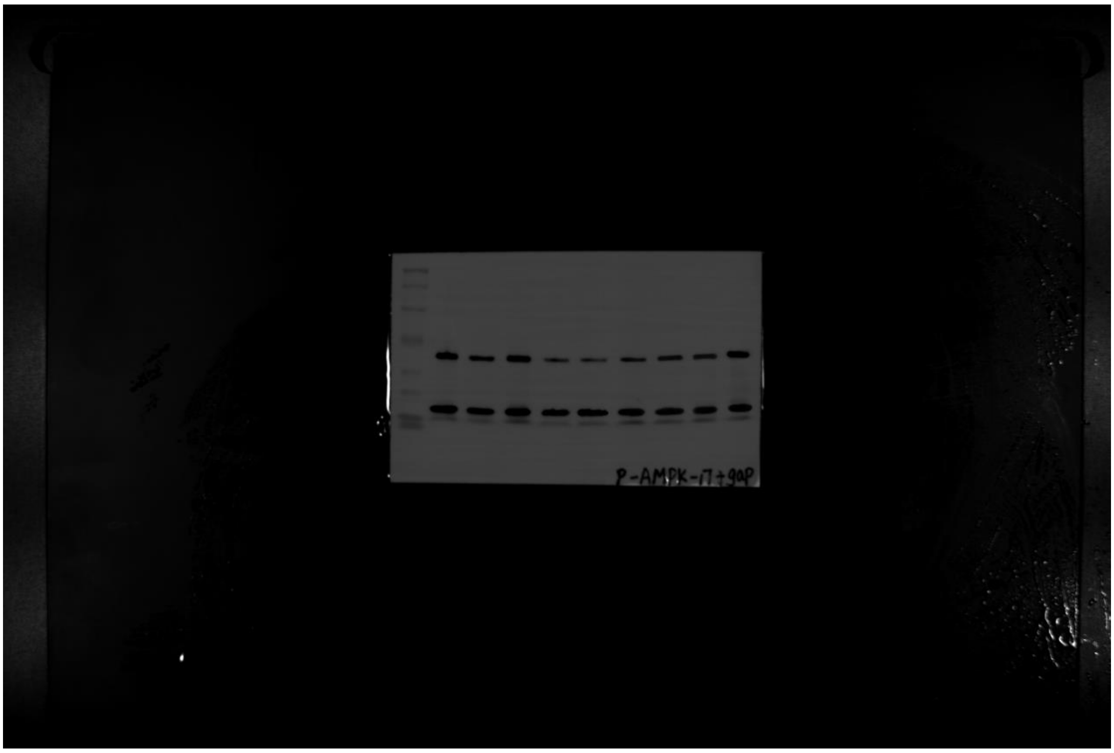

Figure 7B p-AMPK

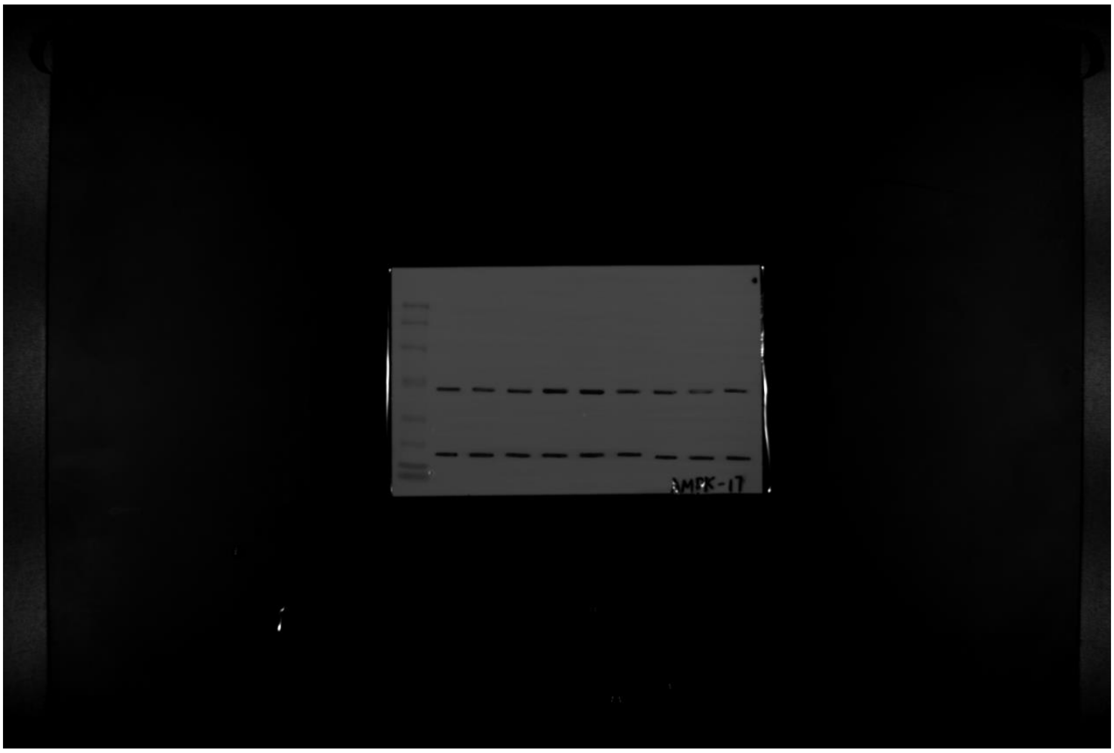

Figure 7B AMPK

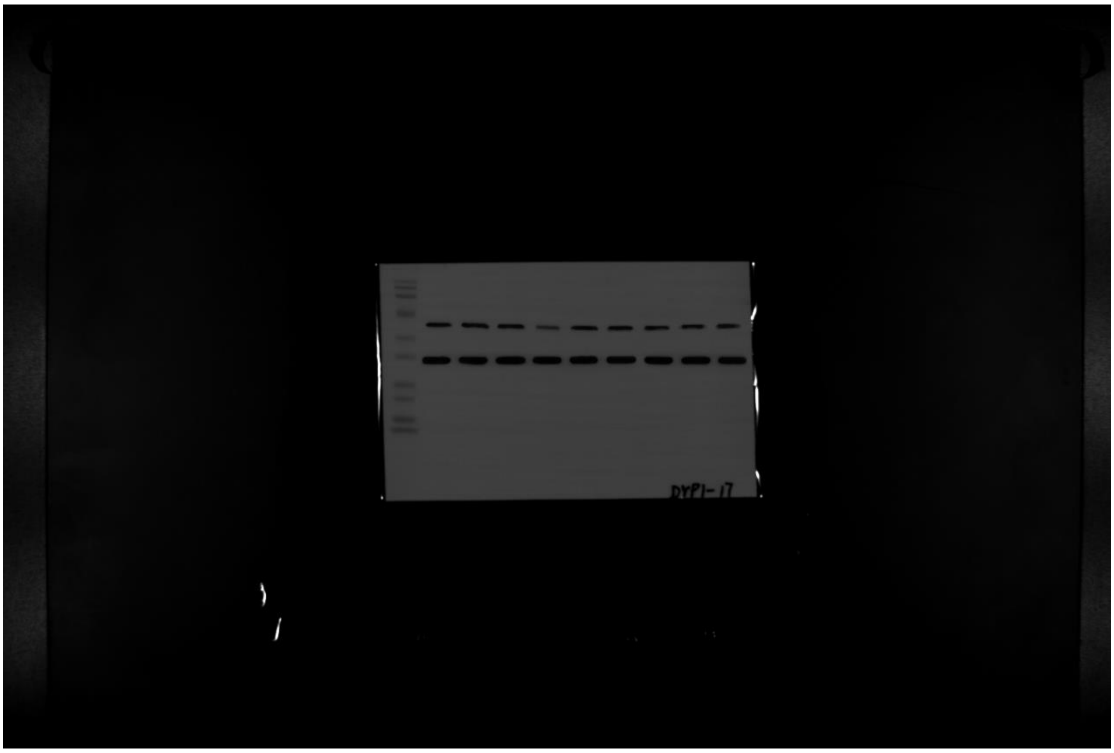

Figure 7B GAPDH

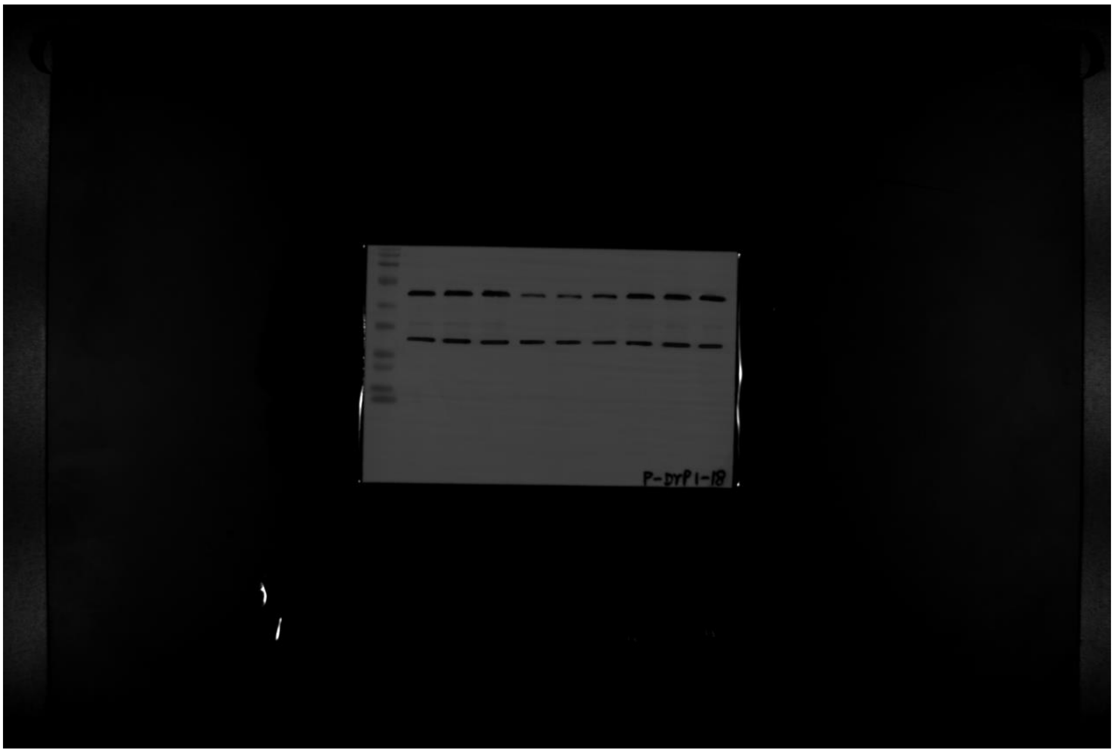

Figure 7B p-DRP1

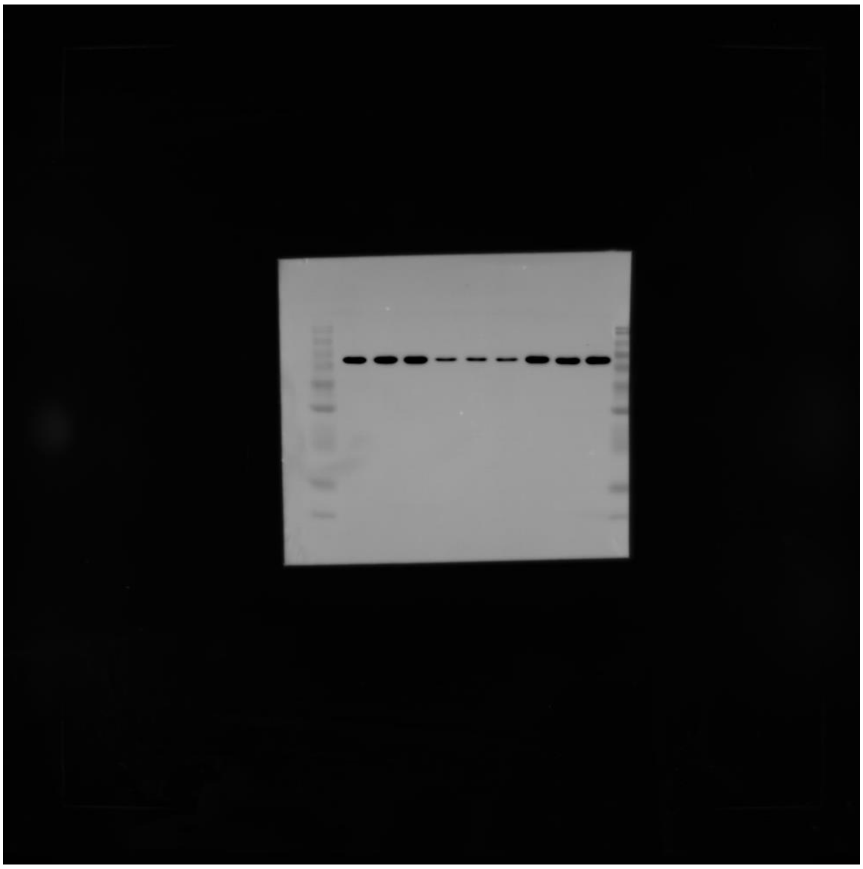

Figure 7B DRP1

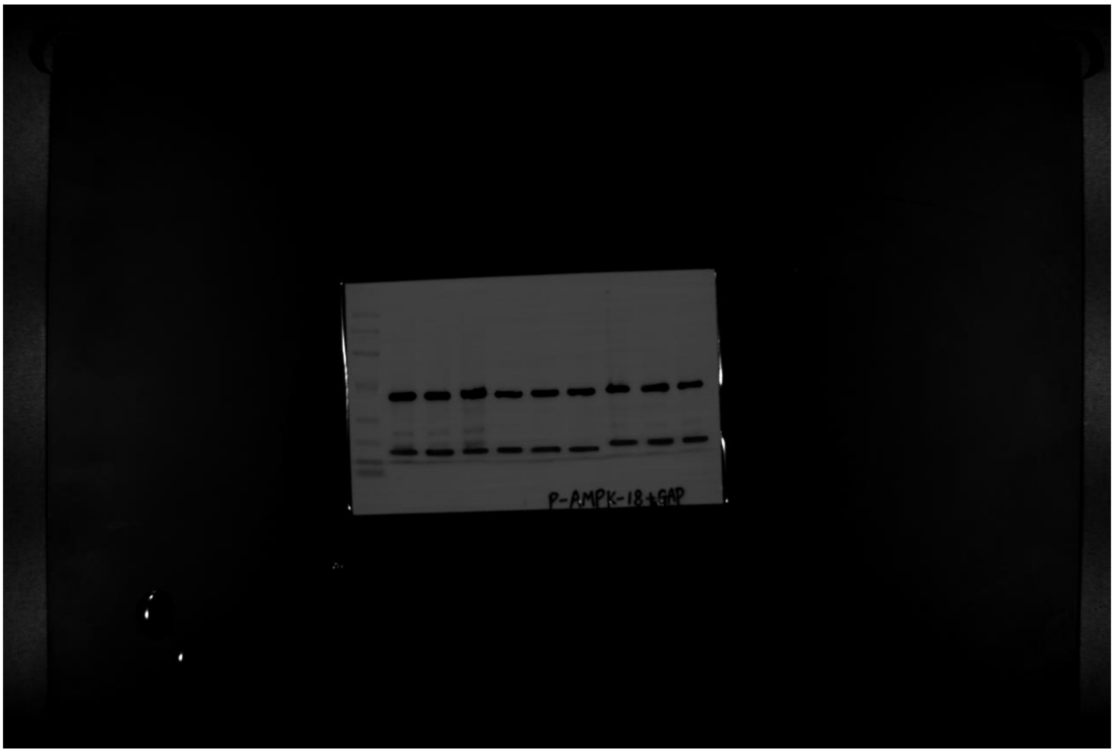

Figure 7B p-AMPK

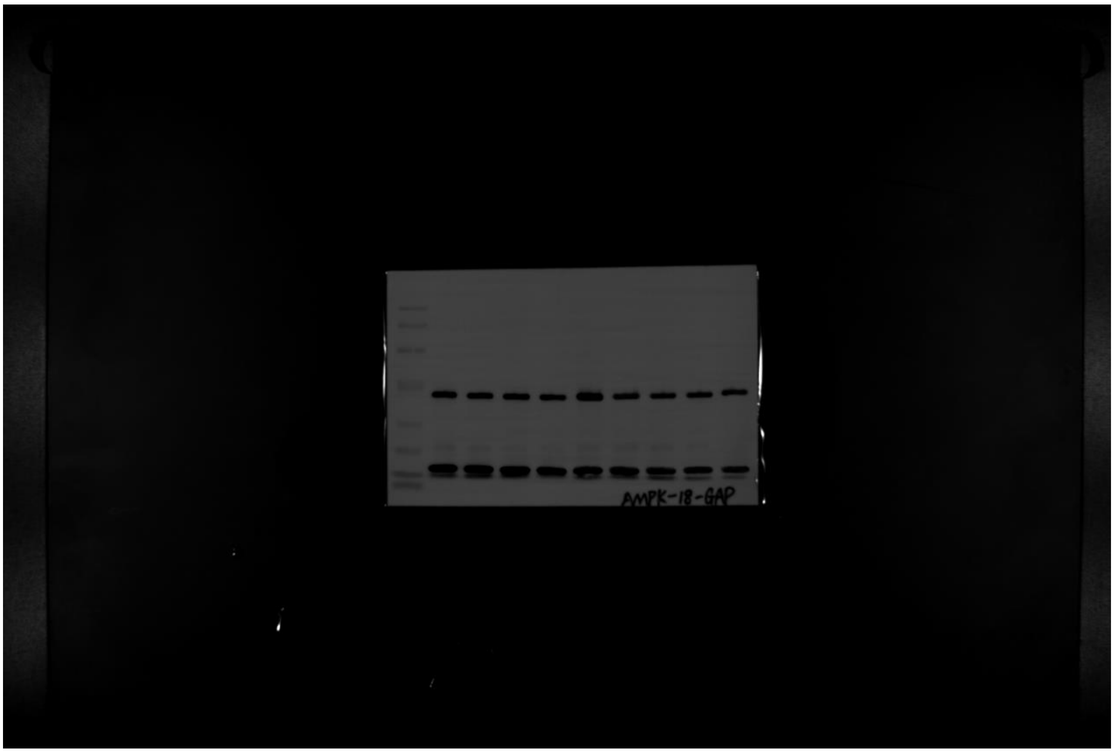

Figure 7B AMPK&GAPDH

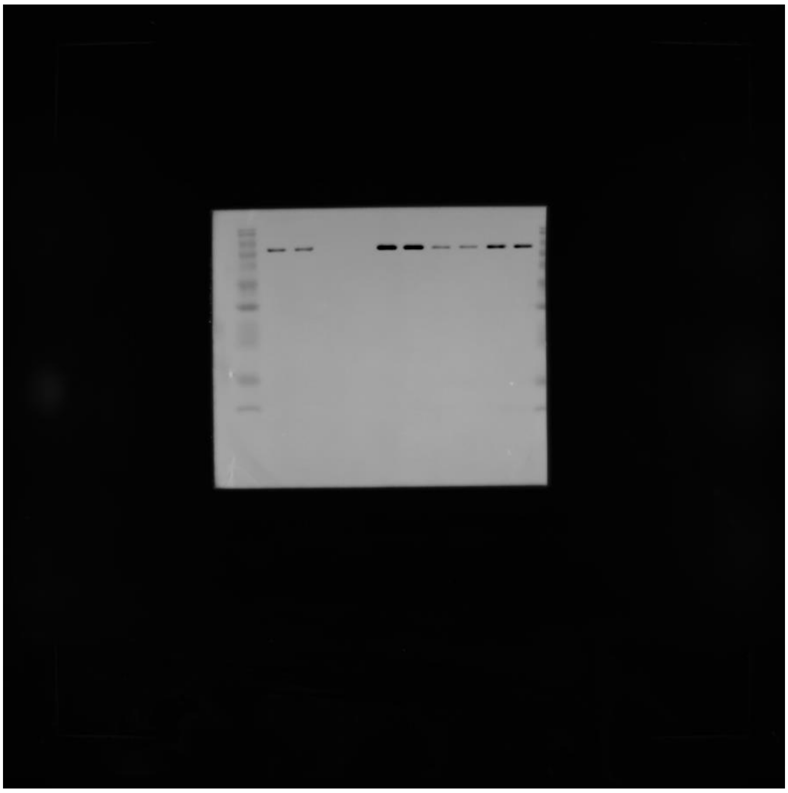

Figure 7C DRP1

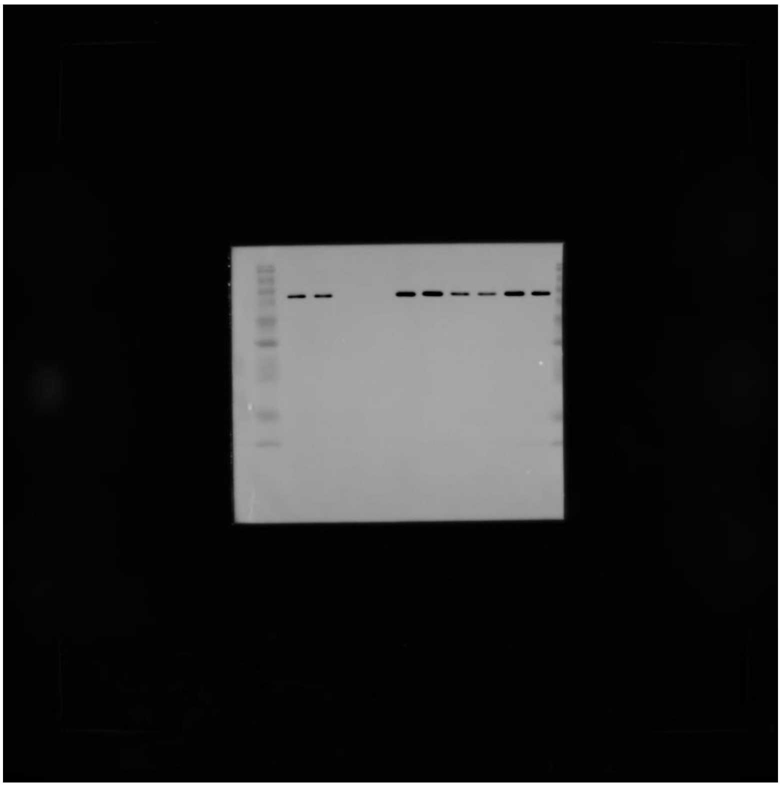

Figure 7C CPT1A

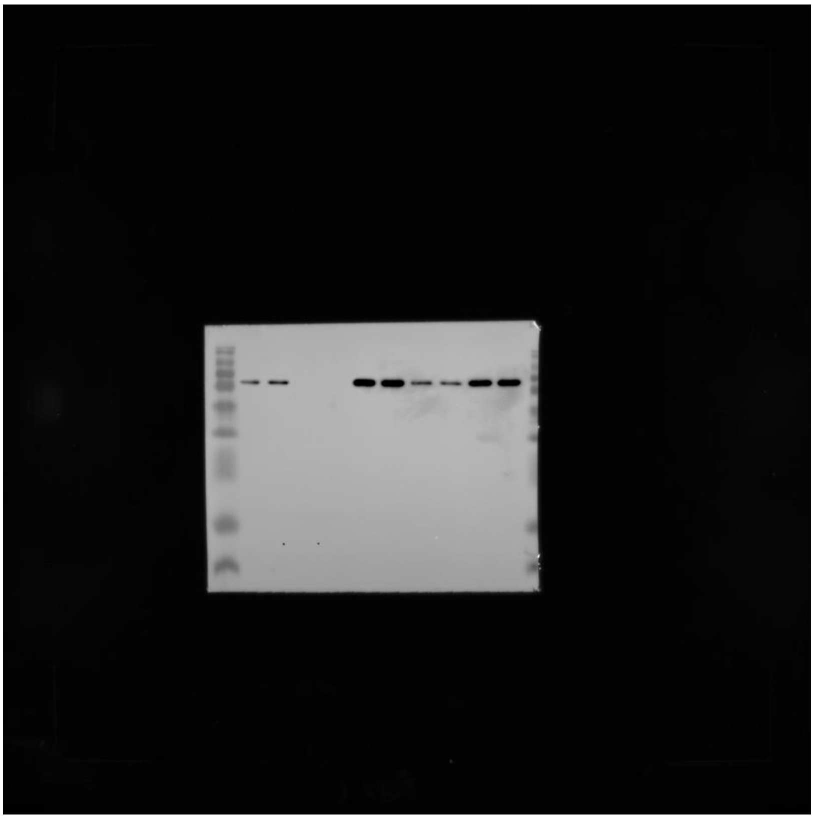

Figure 7C CPT1A

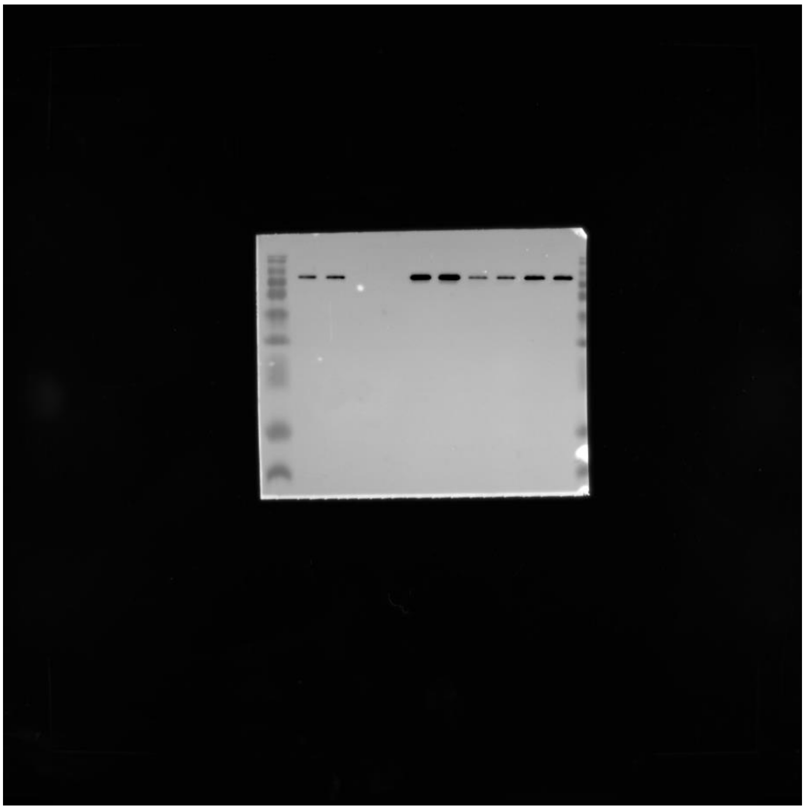

Figure 7C DRP1

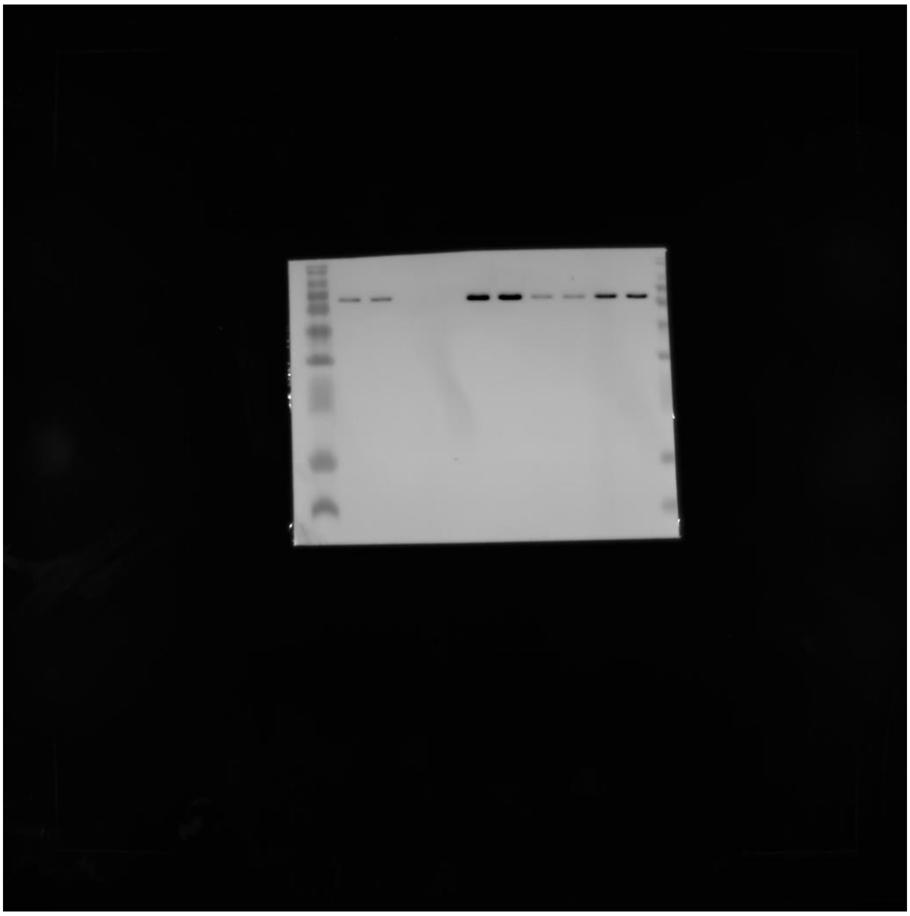

Figure 7D DRP1

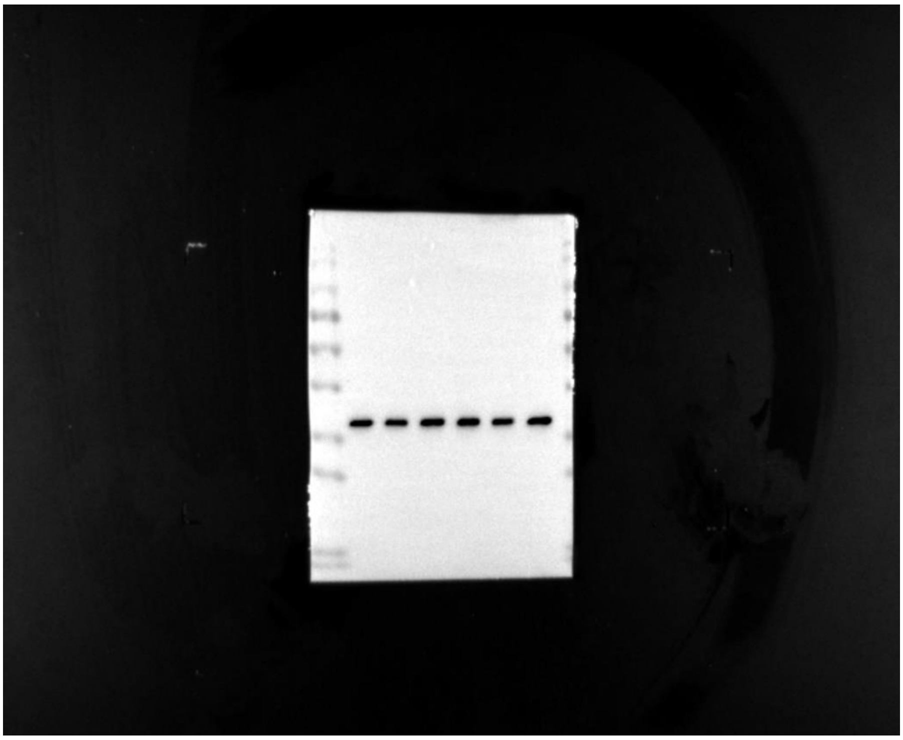

Figure 7D GAPDH

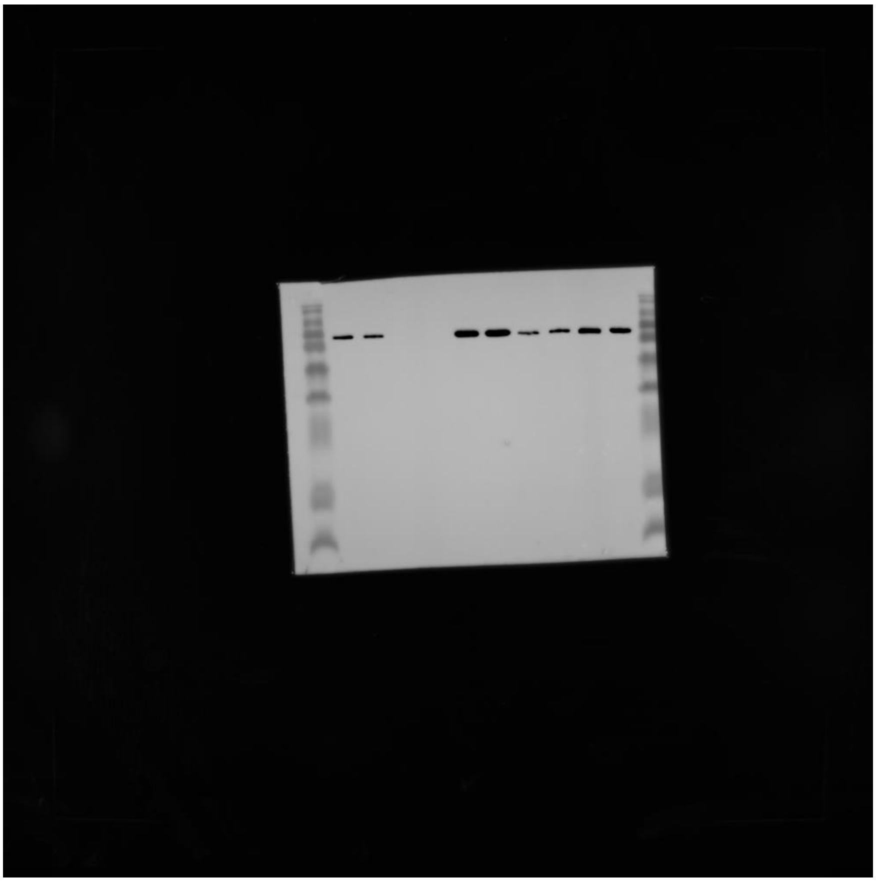

Figure 7D DRP1

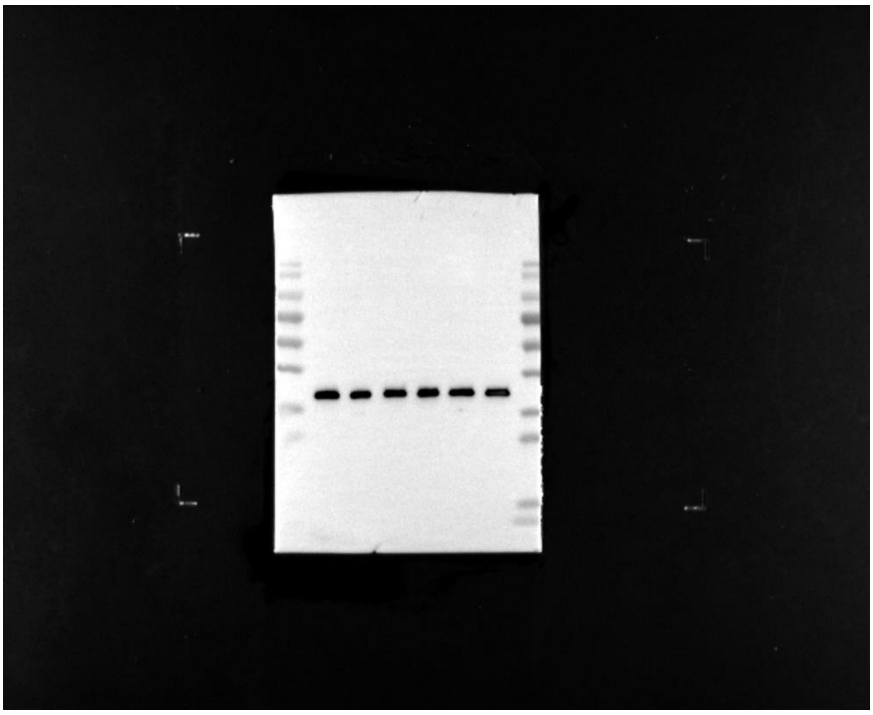

Figure 7D GAPDH
